# Transposable element islands facilitate adaptation to novel environments in an invasive species

**DOI:** 10.1038/ncomms6495

**Published:** 2014-12-16

**Authors:** Lukas Schrader, Jay W. Kim, Daniel Ence, Aleksey Zimin, Antonia Klein, Katharina Wyschetzki, Tobias Weichselgartner, Carsten Kemena, Johannes Stökl, Eva Schultner, Yannick Wurm, Christopher D. Smith, Mark Yandell, Jürgen Heinze, Jürgen Gadau, Jan Oettler

**Affiliations:** 1 Institut für Zoologie, Universität Regensburg, 93053 Regensburg, Germany; 2 Department of Biomolecular Engineering, University of California at Santa Cruz, Santa Cruz, California 95064, USA; 3 Eccles Institute of Human Genetics, University of Utah, Salt Lake City, Utah 84112, USA; 4 Institute for Physical Sciences and Technology, University of Maryland, College Park, Maryland 20742, USA; 5 Institute for Evolution and Biodiversity, Westfälische Wilhelms-Universität, 48149 Münster, Germany; 6 Department of Biosciences, University of Helsinki, 00014 Helsinki, Finland; 7 School of Biological and Chemical Sciences, Queen Mary University of London, London E1 4NS, UK; 8 Department of Biology, San Francisco State University, San Francisco, California 94132, USA; 9 Utah Center for Genetic Discovery, University of Utah, Salt Lake City 84112, USA; 10 School of Life Sciences, Arizona State University, Tempe, Arizona 85287, USA

## Abstract

Adaptation requires genetic variation, but founder populations are generally genetically depleted. Here we sequence two populations of an inbred ant that diverge in phenotype to determine how variability is generated. *Cardiocondyla obscurior* has the smallest of the sequenced ant genomes and its structure suggests a fundamental role of transposable elements (TEs) in adaptive evolution. Accumulations of TEs (TE islands) comprising 7.18% of the genome evolve faster than other regions with regard to single-nucleotide variants, gene/exon duplications and deletions and gene homology. A non-random distribution of gene families, larvae/adult specific gene expression and signs of differential methylation in TE islands indicate intragenomic differences in regulation, evolutionary rates and coalescent effective population size. Our study reveals a tripartite interplay between TEs, life history and adaptation in an invasive species.

## Abstract (web summary)

Genetic variation is key to species evolution. Here the authors sequence two phenotypically distinct populations of the ant *Cardiocondyla obscurior*, and find accumulations of transposable elements correlating with genetic variation that may have a role in differentiation, adaptation and speciation.

## Graphical abstract

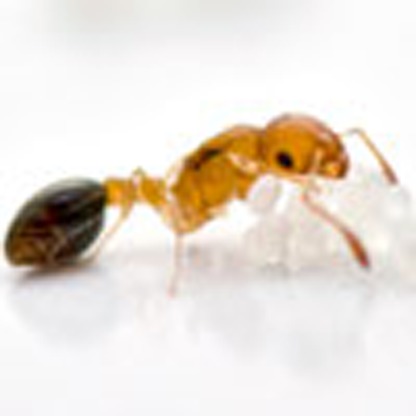

Depletion of genetic variation is detrimental to species evolution and adaptation^1^. Low genetic and phenotypic variation is common in founder populations, where only one or a few genotypes are isolated from a source population. Under such conditions, reduced effective population size (N_e_) should decrease selection efficiency and increase genetic drift, resulting in only weak selection against mildly deleterious alleles which can thus accumulate^2^. These effects should be even stronger in inbreeding species^3^ and taxa with generally low N_e_ such as social insects^4^. Despite these constraints on adaptive evolution, many inbred or selfing species thrive and are able to invade novel habitats. This raises the question of how genetic variation as the raw material for adaptation is generated in such systems.

Single-nucleotide substitutions are an important factor in adaptation^5^ and species diversification^6^,^7^. However, other structural and regulatory units, such as transposable elements (TEs) and epigenetic modifications, may act as drivers in adaptation and evolution^8^. TEs play a particularly vital role in genome evolution^9^ and recurringly generate adaptive phenotypes^10^,^11^,^12^,^13^ primarily through (retro-)transposition^14^, and secondarily through ectopic recombination and aberrant transposition^15^.

The invasive, inbreeding ant *Cardiocondyla obscurior* (Fig. 1) provides a suitable model to study how species adapt to novel habitats in spite of constraints imposed by invasion history, life history or both. Originally from Southeast Asia, *C. obscurior* has established populations in warm climates around the globe from founder populations that presumably consisted of only one or a few inbred colonies, each with a few reproductive queens and several dozen sterile workers. In this species, related wingless males and females (queens) mate within the colony, after which queens leave the colony with a group of workers to find a new nest nearby. While greatly reducing the extent of gene flow between colonies, this behaviour enables sexual reproduction within the same colony and allows single founder colonies to rapidly colonize novel habitats. At the same time, the combination of prolonged inbreeding with severe genetic bottlenecks strongly reduces N_e_ in this species. Under such conditions, genetic drift is predicted to drastically deplete genetic variation, thus leaving little for selection to act on.

Here we explore the genomes of *C. obscurior* from two invasive populations (Brazil BR and Japan JP) to identify signatures of divergence on a genomic level and to determine how the species can rapidly adapt to different habitats. We find clear phenotypic differences between the populations and strong correlation between accumulations of TEs (‘TE islands’) and genetic variation. Our results suggest that TE islands might function as spring wells for genetic diversification in founder populations of this invasive species. The distinct organization of TE islands, their gene composition and their regulation by the genome adds compelling evidence for the role of TEs as players in differentiation, adaptation and speciation.

## Results

### Phenotypic differences between BR and JP lineages

Colonies from the two populations contained similar numbers of workers (Mann–Whitney *U*-test=778.5, *Z*=−0.634, *P*=0.526; BR: median=28, quartiles 21.75 and 51.25, *n*=27 colonies; JP: median=29, quartiles 16 and 47, *n*=64), but queen number was higher in Japan (Mann–Whitney *U*-test=501, *Z*=−3.084, *P*<0.003; BR: 5 queens, quartiles 3, 8; JP: median=10, quartiles 4 and 19). Body sizes of queens and workers from BR were significantly smaller than in JP individuals, yet wingless males did not differ in any of the measured characters (see Supplementary Information).

In ants, cuticular chemical compounds play a particular prominent role in kin recognition, which is crucial for species integrity but on a deeper level also a requirement for the maintenance of altruism^16^. Analysis of cuticular compound extracts from BR and JP workers showed that compound composition differed significantly between the two lineages (multivariate analysis of variance: df=2, F=10.33, *R*^2^=0.39, *P*<0.001) and samples were classified correctly according to population of origin in 83.3% of cases (Supplementary Table 1; Supplementary Fig. 1).

The lineages also differed in behaviour, with BR colonies being significantly more aggressive towards both workers and queens from their own lineage, while JP colonies more readily accepted JP workers and queens (P_Workers_ JPxJP versus BR × BR*=*0.000296, P_Queens_ JP × JP versus BR × BR=7.98e−07, Supplementary Fig. 2). Confronted with individuals from the other lineage, BR colonies were as aggressive as in within-population encounters (P_Workers_ BR × JP versus BR × BR*=*0.39, P_Queens_ BR × JP versus BR × BR=0.94), while JP colonies were again significantly less aggressive (P_Workers_ JP × BR versus BR × BR*=*0.000131, P_Queens_ BR × JP versus BR × BR=1.23e−07). Testing discrimination against workers of another ant species, *Wasmannia auropunctata*, evoked similarly high aggressive responses in both lineages, suggesting that the BR and JP populations do not generally differ in their aggressive potential.

### The *C. obscurior* genome is compact and rich in class I TEs

Using MSR-CA version 1.4, we produced a 187.5-Mb draft reference genome based on paired-end sequencing of several hundred diploid females (454 Titanium FLX sequencing) and a 200-bp library made from five haploid males (Illumina HiSeq2000; Supplementary Table 2), all coming from a single Brazilian colony. Automatic gene annotation using MAKER version 2.20 (ref. 17) was supported by 454 RNAseq data of a normalized library made from a pool of all castes and developmental stages. We filtered the assembly for prokaryotic scaffolds and reduced the initial 11,084 scaffolds to 1,854 scaffolds, containing all gene models and a total of 94.8% (177.9 Mb) of the assembled sequence. The genome can be accessed under antgenomes.org/ and hymenopteragenome.org.

The final gene set contains 17,552 genes, of which 9,552 genes have a known protein domain as detected by IPRScan (www.ebi.ac.uk/interpro/), and falls within the range of recent estimates for eight other sequenced ant species^18^,^19^,^20^,^21^,^22^,^23^,^24^,^25^,^26^. Of all genes, 72.5% have an annotation edit distance of less than 0.5, which is consistent with a well-annotated genome^27^ (Supplementary Table 3).

**Figure 2:**
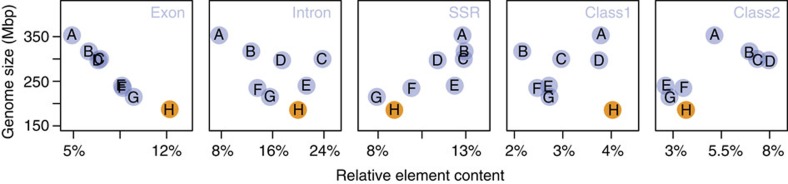
Assembly size in Mbp plotted against the relative proportion of exons, introns and different repetitive elements. The analysed genomes show a negative correlation between relative exon but not intron content. Genome size is positively correlated with relative short simple repeat but not class I and II TE content. A, *S. invicta*; B, *A. cephalotes*; C, *A. echinatior*; D, *H. saltator*; E, *C. floridanus*; F, *P. barbatus*; G, *L. humile*; H, *C. obscurior*.

The *C. obscurior* genome is the smallest so far sequenced ant genome^18^,^19^,^20^,^21^,^22^,^23^,^24^,^25^,^26^. Although there is no physical genome size estimate for *C. obscurior*, assembled sequences and physical estimates are tightly correlated in seven ant genomes (LM in R: *R*^2^=0.73, F_1, 5_=13.7, *P*=0.014, from ref. 28), suggesting that *C. obscurior* has the smallest genome reported so far for an ant species 29. Overall, the draft genome size of the analysed sequenced ants is negatively correlated to relative exon content (GLM in R: df=6, F=150.55, *P*<0.001) but not to relative intron content (df=5, F=0.65, *P*=0.460; Fig. 2), indicative of stabilizing selection on coding sequence. In contrast, intron size distribution is diverse between ant genomes and is not correlated with genome size (Supplementary Fig. 3; Supplementary Table 4).

We used a custom pipeline (see Supplementary Information) to identify simple repeats, class I retrotransposons and class II DNA transposons in *C. obscurior*, seven ant genomes (*Acromyrmex echinatior* (*Aech*), *Atta cephalotes* (*Acep*), *Solenopsis invicta* (*Sinv*), *Linepithema humile* (*Lhum*), *Pogonomyrmex barbatus* (*Pbar*), *Harpegnathos saltator* (*Hsal*), *Camponotus floridanus* (*Cflo*)), the parasitic wasp *Nasonia vitripennis* (*Nvit*) and the honeybee *Apis mellifera* (*Amel*). Across the analysed ants, genome size is significantly correlated with relative simple repeat content (lm, *R*^2^=0.66, F=11.83, *P*=0.014; Fig. 2) but not with class I and class II TE content. However, it appears that the larger genomes contain more relative class II sequence. Relative class I retrotransposon content was highest in *C. obscurior* (7.6 Mb, 4.31%, Supplementary Fig. 4) and in particular, many class I non-LTR retrotransposons (for example, 14 types of LINEs) and several types of LTR transposons (Ngaro, Gypsy, DIRS and ERV2), TIR elements (for example, hAT, MuDR, P) and Helitrons are more abundant in *C. obscurior* (Supplementary Table 5).

### Genomic signatures of an inbred lifestyle

On the basis of TE content calculations for 1 and 200 kb sliding windows, we identified 18 isolated ‘TE islands’ located in ‘LDR’ (low-density regions) in the *C. obscurior* genome. These TE islands were defined as containing TE accumulations in the 95–100% quantile within scaffolds over 200 kb (87 scaffolds, representing 96.02% or 170.8 Mb of the assembly). In total, TE islands cover 12.78 Mb of sequence (7.18% of total sequence) and range between 0.19 and 1.46 Mb in size. The TE islands contain 27.54% (4.92 Mb) of the assembly-wide TE sequence (17.87 Mb), 6.6% of all genes (1,160), and have reduced exon content (TE islands 87.0 exon bp kb^−1^, LDRs 124.5 exon bp kb^−1^). Note that some larger scaffolds contain more than one TE island.

**Figure 3:**
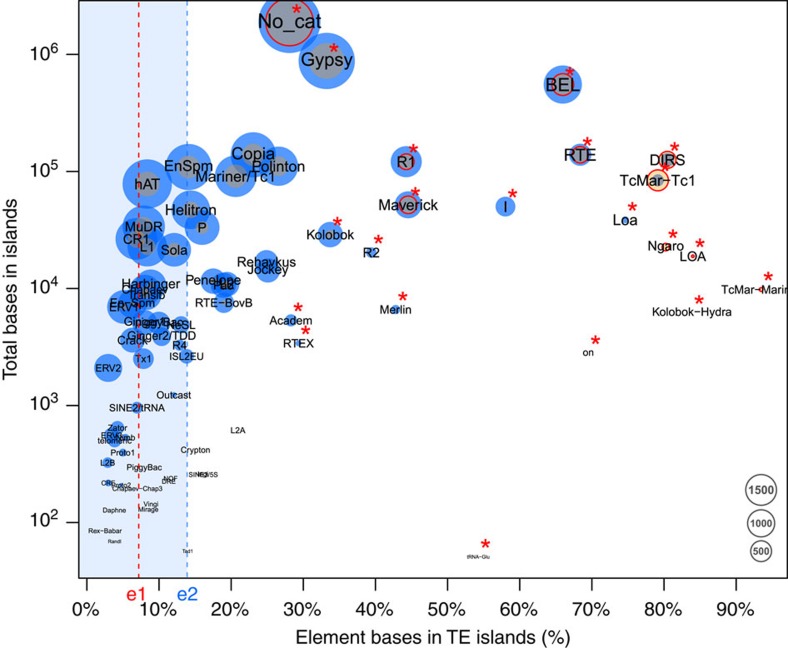
The proportion of bases annotated in TE islands in *C. obscurior* against the log-scaled total base count in TE islands for each TE superfamily. Point size is relative to the copy number of the respective element found in TE islands (orange) and in LDRs (blue). Red circles indicate superfamilies with significantly higher frequency in TE islands than other superfamilies. Superfamilies with a significantly higher base count in TE islands are denoted by a red asterisk. e1: Percentage of the genome contained in TE islands (7.18%), e2: median across all types of TEs (13.89%).

**Figure 4:**
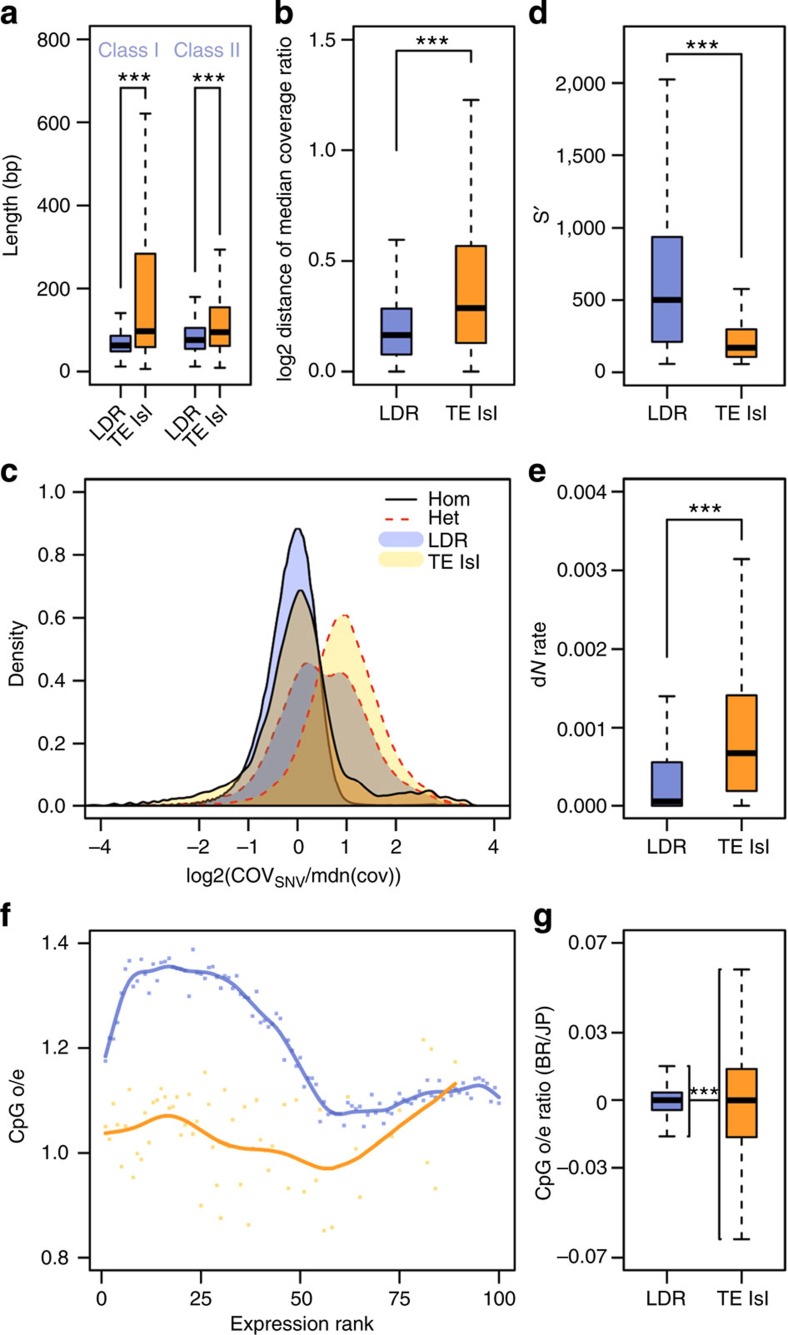
Quantitative measures on the divergence of TE islands and LDRs. (**a**) Length polymorphism for Class I and Class II TEs in LDRs (blue) and TE islands (orange). *U*-tests, *n*_LDR_=54,950, *n*_TE_=6,466 for class I and *n*_LDR_=59,054, *n*_TE_=6,813 for class II. (**b**) Deviations from the median coverage ratio calculated for 1 kb windows in LDRs (blue) and TE islands (orange). *U*-test, *n*_LDR_=157,296; *n*_TE_=12,165. (**c**) Log2-scaled density plots of the coverage for all homozygous (solid black lines) and heterozygous SNV (dotted red lines) calls divided by the median coverage (orange, calls within TE islands; blue, calls in LDRs). Coverage at homozygous calls is not different from the median overall coverage, neither in TE islands nor in LDRs. The shift for heterozygous SNV calls within TE islands shows that most calls result from diverging duplicated loci. The bimodal distribution for heterozygous calls in other genomic regions suggests two distinct populations of SNV calls, that is, true heterozygous loci (first peak) and diverging sequence in duplicated loci (second peak). (**d**) Bit scores for genes in LDRs (blue) and TE islands (orange) retrieved by BLASTx against annotated proteins from seven ant genomes. *U*-test, *n*_LDR_=12,065; *n*_TE_=902. (**e**) Rates of non-synonymous substitutions (calculated as dN/(dN+dS)) in LDR (blue) and TE island genes (orange). *U*-test, *n*_LDR_=6,806; *n*_TE_=423. (**f**) Exon-wide CpG o/e values were plotted against the expression rank from 0 (least expressed) to 100 (most expressed) genes for LDRs (blue) and TE islands (orange). (**g**) Calculated ratios (BR/JP) for exon CpG o/e values in LDRs (blue) and TE islands (orange). F-test, *n*_LDR_=16,379; *n*_TE_=1,159. (***P<0.0001, boxplots show the median, interquartile ranges (IQR) and 1.5 IQR.).

Retroelements of the superfamilies BEL/Pao, DIRS, LOA/Loa, Ngaro, R1/R2 and RTE as well as DNA transposons of the superfamilies Academ, Kolobok-Hydra, Maverick, Merlin, on and TcMar-Mariner/-Tc1 populate TE islands with significantly higher copy numbers than other elements (Fisher’s exact test, false discovery rate <0.05, Fig. 3, Supplementary Table 6). Furthermore, both class I and class II elements show a length polymorphism, with elements in TE islands being significantly longer compared with elements in LDRs (*U*-tests, *W*=109089018, *P*<2e−16 for class I and *W*=152340067, *P*<2e−16 for class II, Fig. 4a, Supplementary Fig. 5).

**Figure 5:**
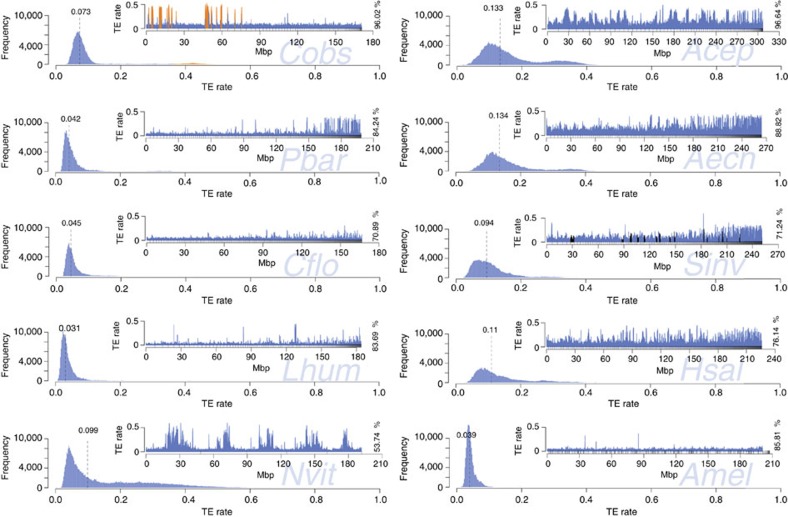
Frequency and distribution (insert plots) of TE content in 200 kb windows. Frequency plots: dashed lines denote median TE content. Distribution plots: different proportions of total draft genome sequence were analysed (in %), depending on assembly quality. Scaffolds are sorted by size, small upward tick marks indicate scaffold boundaries. For *C. obscurior*, regions defined as TE islands are coloured in orange. For *S. invicta*, scaffolds mapping to a non-recombining chromosomal inversion^73^ are shown in black. For *A. mellifera*, scaffolds were sorted according to linkage group.

We also assessed the genome-wide TE distributions for seven published ant genomes, *Amel* v4.5 and *Nvit* v2.0 (Fig. 5). The smaller ant genomes (*Pbar*, *Lhum* and *Cflo*) and *Amel* are similar in TE sequence distribution. In contrast, the larger genomes (*Aech*, *Acep*, *Sinv* and *Hsal*) are more variable, have higher median TE content and a much broader and tailed TE frequency distribution with longer stretches of high or low TE content. The genome of *C. obscurior* is distinct from the other ant genomes, with low TE content in LDRs but exceptional clustering with high TE densities in TE islands. The genome of the inbred wasp *N. vitripennis* contains regions with up to 60% TE content that are surrounded by LDRs containing much less TE sequence (~10%), resembling the pattern observed in *C. obscurior*.

### TE islands diverge faster than LDRs in the two populations

**Figure 6:**
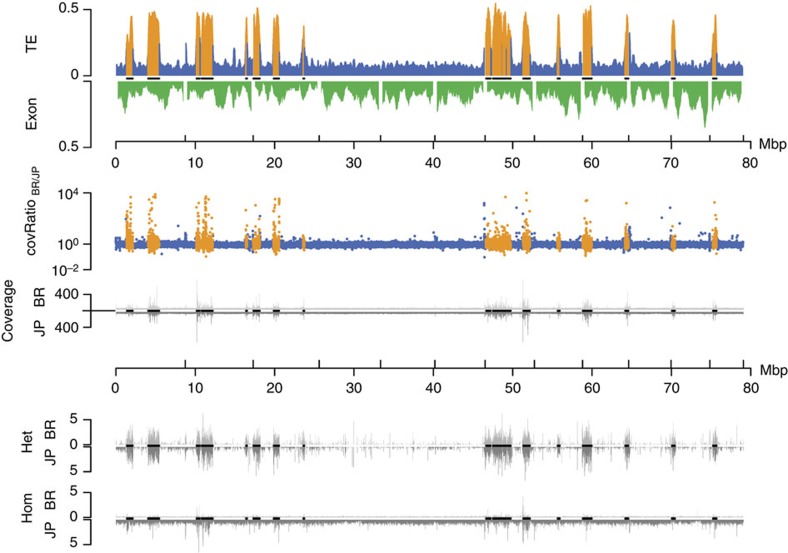
Genomic divergence and subgenomic structure of the 12 largest *C. obscurior* genome scaffolds (including all 18 TE islands). High TE content in TE islands correlates with deviations from the average coverage ratio, very high absolute coverage in both lineages and high numbers of SNV calls. First track: relative TE (blue and orange within TE islands) and exon content (green) per 200 kb. Second track: coverage ratio BR/JP (blue and orange within TE islands). Third track: absolute coverage for BR (top) and JP (bottom). Fourth track: heterozygous SNV calls per kb in BR (top) and JP (bottom) relative to the reference genome. Fifth track: homozygous SNV calls per kb in BR (top) and JP (bottom) relative to the reference genome. Black lines on *x* axes indicate localization of TE islands.

We mapped ~140 Gb of genomic DNA Illumina reads (~60 × coverage for each population) from pools of 30 (BR) and 26 (JP) male pupae, respectively, against the reference genome (BWA; bio-bwa.sourceforge.net) and analysed the local coverage ratio to detect genetic divergence. Deviations from the mean coverage ratio (Fig. 6) are in part caused by sequence deletions, insertions and duplications^30^. Such variations are particularly frequent in TE islands (Figs 4b and 6), suggesting accelerated divergence within islands (median deviation from mean coverage ratio: 0.288 in TE Islands, 0.163 in LDRs; *U*-test, *W*=640300902; *P*<2e−16).

We retrieved SNV (single-nucleotide variants) calls using consensus calls from samtools (samtools.sourceforge.net) and the GATK (broadinstitute.org/gatk/). Although TE islands only comprise 7.18% of the genome, they combine 15.59% (86,236 of 553,052) of all SNV calls. Given that we sequenced haploid males from highly inbred lineages, heterozygous SNVs should be rare. A large fraction of heterozygous SNVs in both lineages are within TE islands (62.95% of 62,879 in BR, 50.52% of 98,353 in JP), while rates of homozygous calls (Fig. 6) are not increased (11.88% of 16,277 in BR, 6.91% of 445,316 in JP). High numbers of false positive heterozygous SNVs calls can arise in duplicated regions that collapsed into a single locus due to misassemblies^31^. Accordingly, such SNVs can be identified by a twofold increase in coverage and in fact mark diverging duplicated loci within the same lineage (Fig. 4c).

Genes in TE islands should also show signatures of accelerated divergence from orthologues if overall sequence evolution is increased in these regions. Indeed, BLASTp searches against seven ant proteomes produced significantly lower bit scores for genes within TE islands when compared with genes in LDRs (Fig. 4d, *U*-test, *W*=120460260, *P*<2e−16). In accordance, SNV annotation revealed higher rates of non-synonymous substitutions between the BR and JP lineage in TE island genes (Fig. 4e, *U*-test, *W*=923754, *P*<2e−16). Surprisingly, however, on average, TE island genes contained less synonymous SNVs than LDR genes (LDR 0.67 kb^−1^, TE island 0.42 kb^−1^, *U*-test, *W*=10743397, *P*<2e−16).

### Copy number variation within and between TE islands

We inspected 512 candidate loci (155 in TE islands) of 1 kb length by plotting the coverage of each lineage relative to SNVs, genes, and TEs at the respective position, to find genes potentially affected by deletion or copy number variation events and compiled a list of 89 candidate genes (Supplementary Table 7). Experimental proof-of-principle was conducted by PCR and Sanger sequencing for two deletion candidates (*Cobs_13563* and *Cobs_01070*) and by real-time quantitative PCR for four duplication candidates (*Cobs_13806*, *Cobs_17872*, *Cobs_13486*, and *Cobs_16853*) (Supplementary Fig. 7). A majority of these genes are located in TE islands (61.8%) and 34 genes show at least weak expression in BR individuals in RNAseq data (see below). The affected genes play roles in processes that may be crucial during invasion of novel habitats, such as chemical perception, learning and insecticide resistance. In particular, four different odorant/gustatory receptor genes show signs of either multiple exon (*Cobs_05921*, *Cobs_13418*, *Cobs_14265*) or whole-gene duplication (*Cobs_17892*). A gene likely involved in olfactory learning, *Cobs_13711*, a homologue to *pst*^32^, also shows signs of duplication. Three genes homologous to fatty acid synthase (FAS) genes, a key step in cuticular odour production, contain partial deletions (*Cobs_16510*, *Cobs_14262*) or duplications (*Cobs_15866*). Furthermore, we found differences in genes associated with insecticide response (*Cobs_00487*, a homologue of *nAChR*α6 (FBgn0032151) (ref. 33) and *Cobs_17834*, coding for a homologue to Cyp4c1 (EFN70878.1) (ref. 34). Other key genes affected are associated with circadian rhythm (*Cobs_17789*, homologue to *per* (FBgn0003068)), caste determination (*Cobs_01070*, with homology to *Mrjp1* (gi406090) (ref. 35), development (*Cobs_17755*, coding for a homologue of VgR (Q6X0I2.1) (ref. 36) and aging (*Cobs_14758*, with homology to *Mth2* (FBgn0045637) (ref. 37).

*De novo* assembly of ~23M Illumina paired-end reads from the JP lineage that could not be mapped to the BR reference genome resulted in 17 contigs after filtering with highly significant BLASTx hits against proteins of other ants, suggesting that these conserved sequences were lost in the BR lineage instead of being gained in the JP lineage. According to functional annotation, among others these contigs code for homologues involved in development (Vitellogenin-like (XP_003689693))^38^, cellular trafficking (Sorting nexin-25 (EGI65030))^39^, immune response (Protein Toll (EGI66069))^38^ and neuronal organization (Peripheral-type benzodiazepine receptor-associated protein 1 (EFN68490))^40^ (Supplementary Table 8).

### Gene composition and regulation of TE islands

Increased TE activity may incur costs to fitness by disrupting gene function. A two-tailed Gene Ontology (GO) enrichment analysis revealed that 59 GO terms associated with conserved processes (for example, cytoskeleton organization, ATP binding, organ morphogenesis) are under-represented in TE islands, while 18 GO terms are enriched (Supplementary Tables 9 and 10). Four of the over-represented terms relate to olfactory receptors (ORs; GO:0004984, GO:0005549, GO:0050911, GO:0007187) and two terms relate to FAS genes (GO:0005835, GO:0016297). The remaining 12 terms most likely relate to TE-derived genes.

Gene body CpG depletion as a result of increased CpG to TpG conversion due to cytosine methylation is a measure for germline methylation (that is, epigenetic regulation) in past generations. In TE island genes, the exon-wide median observed/expected (o/e) CpG ratio is significantly lower than in other genes (*t*-test, TE island genes: 1.05, LDR genes: 1.20, *P*<1e−16). However, both sets of genes show strikingly different correlations of expression and o/e CpG values (Fig. 4f). For LDR genes, o/e CpG values are high in moderately expressed genes and low in highly expressed genes. In contrast, in TE islands, weakly to moderately expressed genes contain less CpG dinucleotides, while highly expressed genes have higher o/e CpG values. To further identify traces of differential regulation of TE islands, we compared the exon o/e CpG values between the lineages by calculating BR/JP ratios for each exon’s o/e CpG values and found higher variance in BR/JP ratios in TE islands than in LDRs (Fig. 4g, F-test, F=0.136, *P*<2e−16, ratio of variances=0.136).

**Figure 7:**
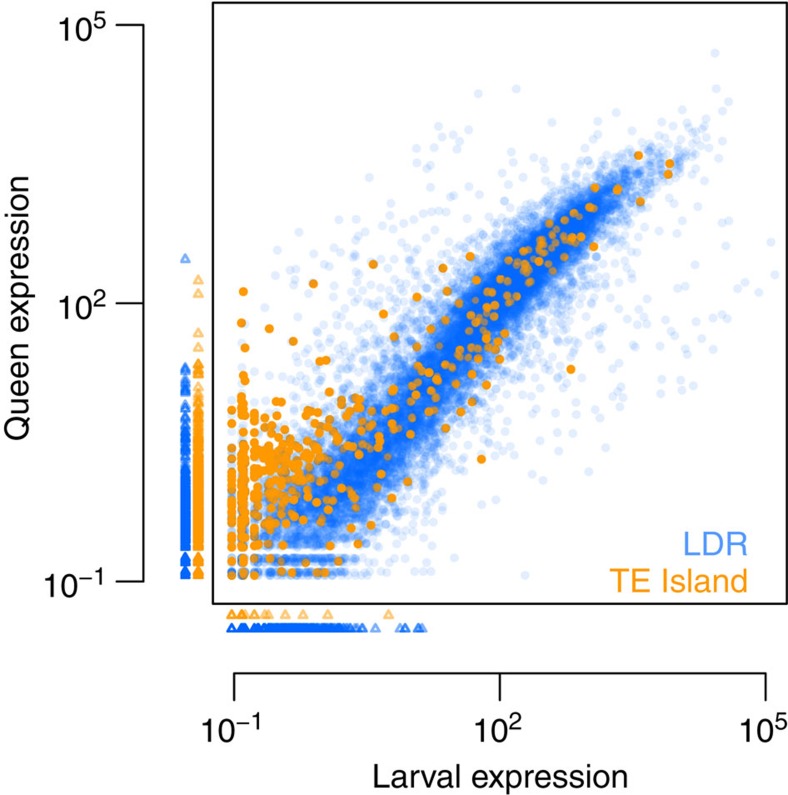
Mean normalized expression in third instar queen larvae and mated adult queens for all Cobs1.4 genes. Small triangles indicate genes with no expression in queens (plotted below the *x* axis) or larvae (plotted left to the *y* axis). Ninety-five TE island genes and 1,382 LDR genes were not expressed at all (orange, TE island genes; blue, LDR genes).

Finally, to assess whether gene expression levels differed between LDRs and TE islands, we generated ~14 and ~17 Gb transcriptomic RNAseq data of seven queens and seven queen-destined larvae (third larval stage), respectively, from the BR lineage. We estimated mean normalized expression values for each gene using DESeq2 (bioconductor.org/packages/release/bioc/html/DESeq2.html), revealing that expression in TE islands was much lower than in LDRs (median expression of all LDR genes=25.45; in TE islands: 0.49; *U*-test, *W*=14461310, *P*<2e−16). While larvae and adult queens did not differ in the expression of LDR genes (median expression in queens=21.16; in larvae=23, 72; *U*-test, *W*=133301709, *P*=0.221), TE island genes were more expressed in adult queens (median expression in queens=0.84; in larvae=0; *W*=1031038, *P*<2e−16; Fig. 7, see Supplementary Fig. 6 for details on differential expression between queen and larvae).

## Discussion

*C. obscurior* is a textbook example for successful biological invasion. Its small size allows for interspecific avoidance, it can rapidly establish colonies in disturbed habitats, and multiple generations per year allow for fast adaptation. While variation in CHCs and body size between the populations point to adaptations to different environments, higher queen number in the JP lineage is likely correlated with reduced intraspecific aggression.

The small genome of *C. obscurior* differs markedly from the other analysed ant genomes in TE distribution and overabundance of several class I subclasses. Importantly, the genome contains low frequencies of TEs in LDRs but well-defined islands with high densities of TEs. In these islands, TEs are on average longer than in LDRs, suggesting overall higher TE activity^41^. Differences in mutation rates and sequence divergence between LDRs and TE islands reveal distinct evolutionary dynamics acting within the *C. obscurior* genome. Moreover, in TE islands, key genes are removed and the majority of genes is less expressed in larvae than adult queens. The non-random distribution of TEs suggests that intragenomic differences in selection efficiency against TEs may have further supported the formation of such locally confined TE accumulations.

Inbreeding can facilitate the accumulation of TEs^3^ and repeated exposure to stress induced by novel environmental conditions can further amplify TE proliferation^42^. Small N_e_ is expected to increase the effects of genetic drift and in turn reduce selection efficiency against mildly deleterious mutations^2^. Under such conditions, local accumulations of TEs might have formed in genomic regions under relaxed selection. Similarly, a reduction in N_e_ in inbred *Drosophila* leads to a shift in the equilibrium between TE proliferation and purifying selection against TEs, thus allowing TEs to accumulate^43^.

How can we explain extensive proliferation and diversification of TEs within islands, but purifying selection against TEs in LDRs? Coalescent effective population size of a genomic region is positively correlated with its recombination frequency and thus the local efficiency of selection and mutation rate^11^. The initial foundation of TE islands could hence be facilitated in genomic regions with low recombination frequency, providing a refugium of relaxed selection for TE insertions. Indeed, elevated rates of non-synonymous substitutions suggest relaxed selection on TE island genes. Increased frequency of DNA repair processes as a consequence of higher DNA transposition frequencies in TE islands should lead to more errors in DNA replication and double strand break repair^44^ in comparison with LDRs. Large-scale mutations on the other hand, such as exon or gene duplications/deletions or gene shuffling, can directly be introduced during TE transposition^45^. TE islands may frequently produce genetic novelty and eventually, by chance, but despite high stochastic drift, adaptive phenotypes, corroborating the view of TEs as genetic innovators.

The list of genes affected by duplications or deletions contains a number of candidates that might be key to the divergence of the lineages. For example, differences in homologues to genes involved in larval development (for example, *Mrjp1*) might explain body-size differences. Two other candidates, *Cobs_00487* and *Cobs_17834*, show homology to genes that are involved in pesticide resistance against Chlorpyrifos and Imidacloprid (*nAChRα6*) and Deltamethrin (*Cyp4c*) in different invertebrate species^46^,^47^,^48^,^49^. Imidacloprid treatment of gall wasp infested *Erythrina variegate* coral trees of the Japan habitat occurred at least once the year before collection of the colonies in 2010 (personal communication S. Mikheyev). In the Brazil habitat, Chlorpyrifos, Deltamethrin and the organophosphate Monocrotophos have routinely been used over the last 10 years (personal communication J.H.C. Delabie).

Furthermore, several within-island genes involved in the production (FAS^50^) and perception (ORs) of chemical cues contained deletions or duplications in one of the lineages. These results suggest that variation in FAS genes may be responsible for diverging CHC profiles in *C. obscurior*^51^, while variation in OR genes affects olfactory perception. Chemosensory neurons express highly sensitive ORs^52^, which are particularly diverse^53^ and under strong selection in ants^54^. Gene loss and duplication in the OR gene family has been significantly frequent^55^ and differences are assumed to be shaped by adaptive processes in response to a species’ ecological niche^56^,^57^. Intriguingly, the diversification of OR genes is thought to be largely caused by gene duplications and interchromosomal transposition^58^, two mechanisms known to be by-products of TE activity. While the distinct patterns of kin recognition and aggressive behaviour in the two lineages of *C. obscurior* may in part be explained by TE-mediated variation in these genes, they also suggest lineage-specific dynamics of the interaction of phenotype and genome evolution. Reduced aggression between colonies in the JP lineage should promote gene flow by exchange of reproductives and thus increase Ne, heterozygosity, and the efficiency of sexual recombination, facilitating the spread of novel arising genotypes. Our findings contrast the view of reduced aggression between colonies of invasive ants^59^, but so far it is unclear whether lineage-specific differences are caused by variation in perception or downstream neuronal processes.

Mechanisms controlling TEs are as old as prokaryotes^9^ and in fact most TEs are epigenetically silenced^45^,^60^, through either methylation, histone modifications^61^ or RNAi^62^. Even though many genes in TE islands are expressed, the overall expression is significantly lower than in LDRs. In line with previous correlations on methylation and expression in eusocial insects^63^,^64^, o/e CpG ratios in *C. obscurior* LDR genes are negatively correlated with expression. However, TE island genes do not follow this trend, in that they are weakly expressed while having low o/e CpG rates. Proximity to TEs can increase gene body methylation^65^, which could explain stronger methylation of TE island genes and thus CpG depletion. Also, relaxed selection in island genes should in general increase fixation frequency of base mutations, including CpG to TpG conversions thus depleting CpG content. Gene expression differences in TE island genes between larvae and adult queens suggest stronger regulation of these potentially disruptive genes during the sensitive developmental phase. Finally, key regulatory genes are under-represented in TE islands. These gene set differences between TE islands and LDRs can either be explained by selection processes, removing vital genes from linkage to TE islands or by selective restriction of TE accumulations to genomic regions devoid of such genes.

The current understanding of TE activity dynamics in genomes is that periods of relative dormancy are followed by bursts of activity, often induced by biotic and abiotic stress, such as exposure to novel habitats. Frequent TE transposition during bursts leads to genomic rearrangements, thus producing new genetic variants and eventually even promoting speciation^66^,^67^,^68^,^69^. TE dynamics can also be strongly affected by mating system^3^,^70^,^71^,^72^, and the life history of *C. obscurior* likely challenges the genomic integrity resulting in genomic regions with over 50% TE content. In conclusion, TE dynamics in *C. obscurior* seem to have shifted from a serial to a parallel mode, where a fraction of the genome is reshaped repeatedly in a continuous burst of TE activity. Strikingly, the inbred parasitoid wasp *N. vitripennis* has similar TE frequency patterns suggesting that similar life history strategies and their consequences on N_e_ and drift can lead to convergent genomic organization. TEs represent a major force in evolution, contributing to the generation of genetic variation especially in species confronted with hurdles like inbreeding or repeated bottlenecks. They furthermore seem to play an important role in the rapid adaption of invasive species to novel environments, making it particularly crucial to understand their origin, function and regulation.

## Methods

Detailed methods and accompanying Supplementary Tables 11 to 16 are available as Supplementary Information online.

### Organisms

Live colonies of *C. obscurior* were collected from aborted fruits on coconut trees (*Cocos nucifera*) in Brazil (collected in 2009) and from bark cavities in coral trees (*Erythrina* sp.) in Japan (collected in 2010). The colonies were transferred to Regensburg and placed in plastered petri dishes. Food (honey-soaked shreds of paper; *Drosophila* or small chunks of *Periplaneta americana*) and water were provided every 3 days and colonies were kept in incubators under constant conditions (12 h 28 °C light/12 h 24 °C dark). All animal treatment guidelines applicable to ants under international and German law have been followed. Collecting the colonies that form the basis of the laboratory population used in this study was permitted by the Brazilian Ministry of Science and Technology (RMX 004/02). No other permits were required for this study.

### *De novo* genome assembly

The reference genome is based on one colony that was kept under strict inbreeding in the lab for four generations before extractions. Whole DNA was extracted with CTAB. We extracted DNA from ~900 ants, which were pooled to be sequenced with 454 technology. Extracts of 5, 10 and 30 Brazilian males and 26 Japanese males, respectively, were used for Illumina libraries.

We generated 200 and 500 bp insert libraries with Illumina’s TruSeq DNA sample preparation kits from 5 μg of total DNA. Quality control and library preparation were carried out by the KFB sequencing centre of the University Regensburg, sequencing runs were performed by Illumina (Hayward, USA) on a HiSeq2000. Quality control, library preparation and sequencing of 8 and 20 kb long paired end libraries (454, Roche) were carried out by Eurofins MWG Operon (Ebersberg, Germany). Extracted DNA was fragmented into the appropriate fragment sizes (8 and 20 kb) using the HydroShear DNA Shearing Device (GeneMachine). Further library preparation was performed according to ‘GS FLX Titanium Paired End Library Prep 20+8 kb Span Method Manual’ before sequencing on a GS FLX Titanium (Roche).

The *de novo* genome assembly was created with MSR-CA version 1.4 open source assembler (University of Maryland genome assembly group at ftp://ftp.genome.umd.edu/pub/MSR-CA/). The MSR-CA assembler combines a deBruijn graph strategy with the traditional Overlap-Layout-Consensus employed by various assembly programmes for Sanger-based projects (Arachne, PCAP, CABOG). The MSR-CA uses a modified version of CABOG version 6.1 for contiging and scaffolding. The combined strategy allowed us to natively combine the short 100 bp Illumina reads and longer 454 reads in a single assembly without resorting to an approach that would require one to assemble each type of data separately and then creating a combined assembly.

### Mapping

For each lineage, we randomly sampled 140 M 100 bp reads from libraries generated from 26 (JP) and 30 (BR) male pupae. Raw reads were parsed through quality filtration and adapter trimming (Trimmomatic v0.22 (usadellab.org/cms/?page=trimmomatic), options: HEADCROP:7 LEADING:28 TRAILING:28 SLIDINGWINDOW:10:10) and mapped against the BR reference genome with BWA (bio-bwa.sourceforge.net) and Stampy v1.0.21 (www.well.ox.ac.uk/project-stampy).

### Variant calling

SNV calling was carried out combining samtools (samtools.sourceforge.net) and the GATK (www.broadinstitute.org/gatk/) retaining only those variants called consistently by both tools. The final variant set of 553 052 SNVs and 67,987 InDels was stored in a single VCF file. SNVs were annotated with SNPeff (snpeff.sourceforge.net) to identify non-synonymous and synonymous substitutions.

### Calculation of sliding windows

One kb windows of different stats (TEs, exons, SNPs, coverage) were calculated for all scaffolds based on GFF, VCF and SAM files. For GFF and VCF files, custom bash and perl scripts were used to calculated TE and exon bases per 1 kb, and variant calls per 1 kb. Coverage per 1 kb was calculated from SAM files, using samtools’ depth algorithm and custom bash and perl scripts. Subsequent processing, calculating of 200 kb sliding windows and plotting of the data was performed with R v3.0.0 (r-project.org).

### Gene expression analysis with RNAseq

We extracted whole RNA with the RNeasy Plus Micro kit (Qiagen). Single end Illumina libraries from amplified RNA (Ovation RNAseq system V2) were generated following the manufacturers protocol (Ovation Rapid Multiplexsystem, NuGEN). Sequencing on an Illumina HiSeq1000 at the in-house sequencing centre (KFB, Regensburg, Germany) generated ~20M 100 bp reads per sample (Supplementary Table 16). Raw reads were filtered for adapter contamination (cutadapt, code.google.com/p/cutadapt/), parsed through quality filtration (Trimmomatic v0.27, options: LEADING:10 TRAILING:10 SLIDING:4:10 MINLEN:15), and mapped against the reference genome using the tophat2 (v2.0.8, ccb.jhu.edu/software/tophat/index.shtml) and bowtie2 (v2.1.0, bowtie-bio.sourceforge.net/bowtie2/index.shtml) package (--b2-sensitive mode, mapping rate ~50%). Gene expression analysis was carried out with DESeq2 (bioconductor.org/packages/release/bioc/html/DESeq2.html), based on count tables produced with HTSeq (www-huber.embl.de/users/anders/HTSeq/doc/overview.html) against the Cobs1.4 MAKER annotation (Supplementary Table 16). Genes were considered to be differentially expressed at a false discovery rate <0.05 and expression values are reported as untransformed base means of read counts per treatment group, after correcting for library size differences (‘size factor normalization’).

## Author contributions

J.O. and L.S. designed the study; J.O., L.S., J.G. J.H. wrote the manuscript; L.S. and J.O. analysed the data; A.Z. was responsible for genome assembly; J.W.K. and C.D.S. were responsible for repeat annotation; D.E., M.Y. and L.S. were responsible for gene prediction; C.K. was responsible for CpG o/e calculation; Y.W. was responsible for data logistics; J.O., L.S., T.W., K.W. and A.K. were responsible for analyzing phenotypic differences; J.S., E.S. and J.O. performed the chemical analyses; all authors read and commented on the manuscript.

## Additional information

**How to cite this article**: Schrader, L. *et al*. Transposable element islands facilitate adaptation to novel environments in an invasive species. *Nat. Commun.* 5:5495 doi: 10.1038/ncomms6495 (2014).

**Accession codes:** Raw sequencing data from this study have been deposited in GenBank/EMBL/DDBJ under the BioProject accession code PRJNA237579.

## Supplementary Material

Supplementary Information Supplementary Figures 1-7, Supplementary Tables 1-16, Supplementary Methods and Supplementary References

## Figures and Tables

**Figure 1:**
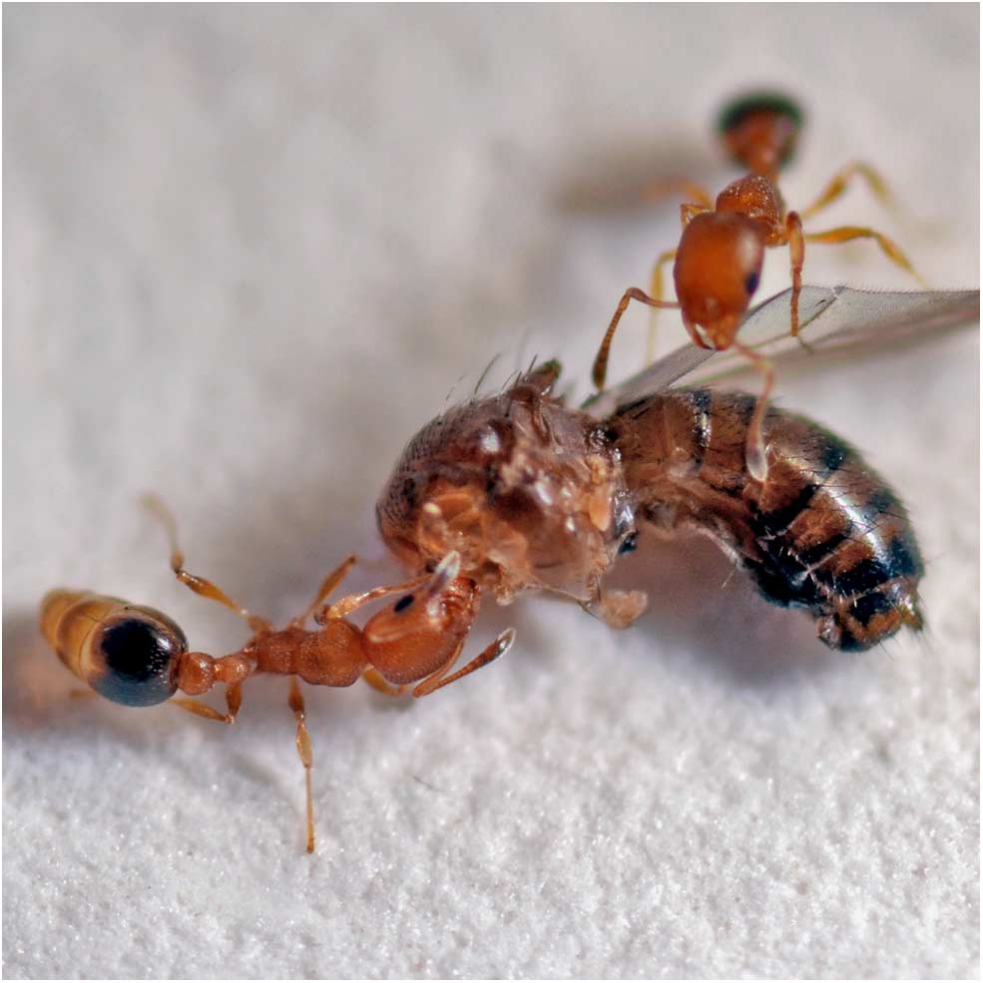
Two workers of *C. obscurior* and the remains of a fly. Hidden in small cavities of plants, the inconspicuous colonies of this species are frequently introduced to new habitats by global commerce. In spite of strong genetic bottlenecks, even single colonies with few reproductive individuals suffice to establish stable populations.
