# Supplementary material for: Transposable element islands facilitate adaptation to novel environments in an invasive species
Source: Nat Commun. 2014 Dec 16;5:5495. doi: 10.1038/ncomms6495 (PMC4284661; doi:10.1038/ncomms6495)
Supplement: Supplementary Information — Supplementary Figures 1-7, Supplementary Tables 1-16, Supplementary Methods and Supplementary References [file ncomms6495-s1.pdf]

Supplementary Figures

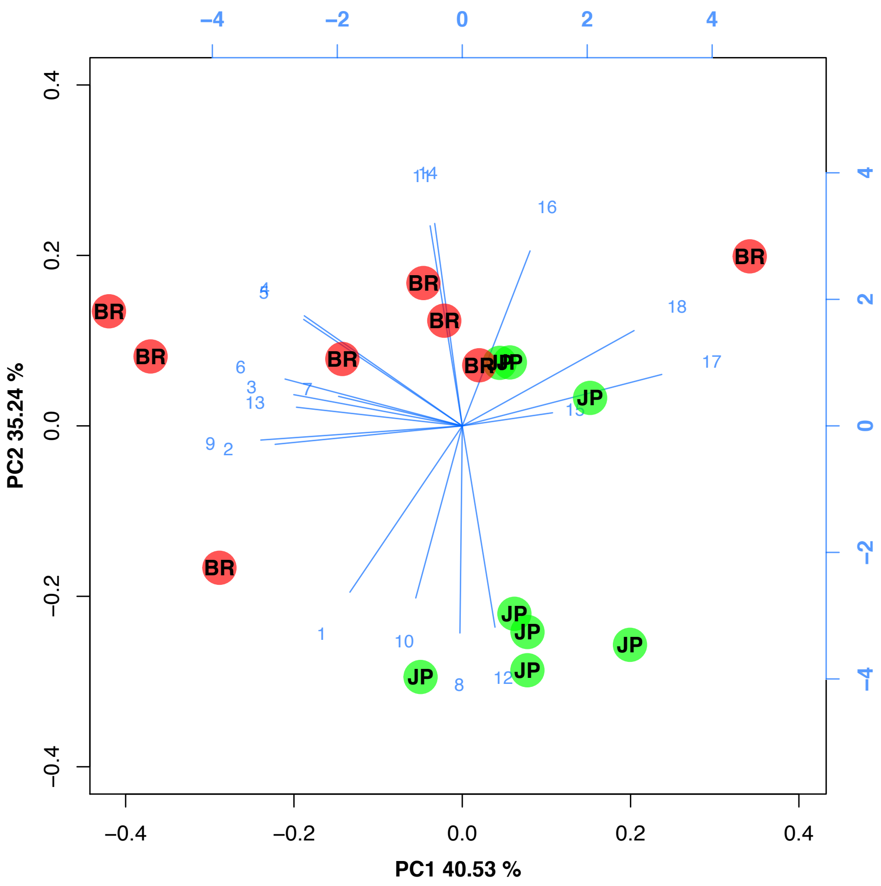

Supplementary Figure 1a: PCA plot of 22 Aitchison-transformed peak-areas

PC1 explains 40.53 % of the variance in the data matrix. Blue lines denote peak number.

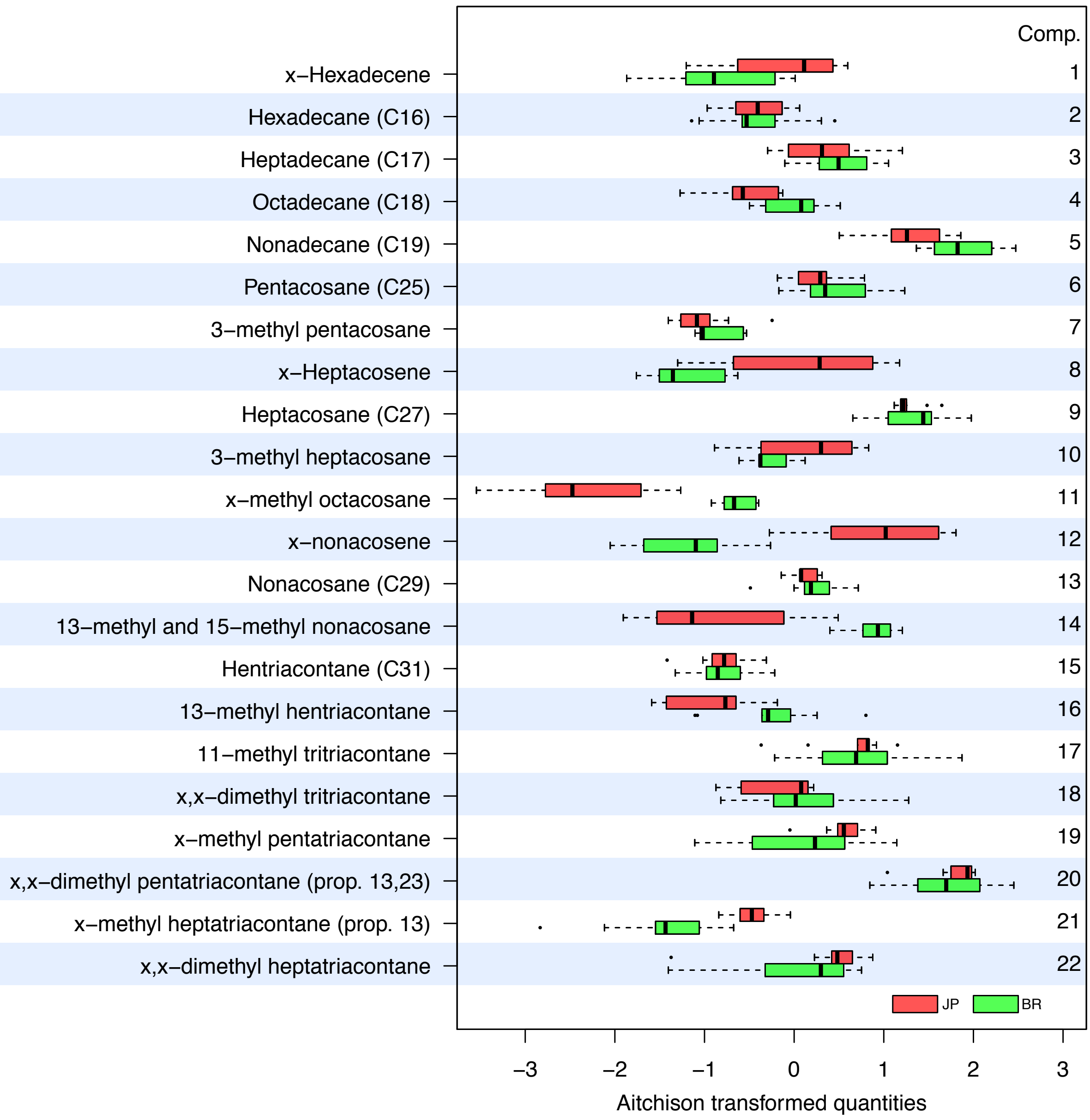

Supplementary Figure 1b: Boxplot of relative compound abundance in each lineage

Boxplot of relative compound abundance in each lineage. 'x' denotes unknown position of the double bond or methyl branch. Compound 14 could not be separated with the GC parameters used.

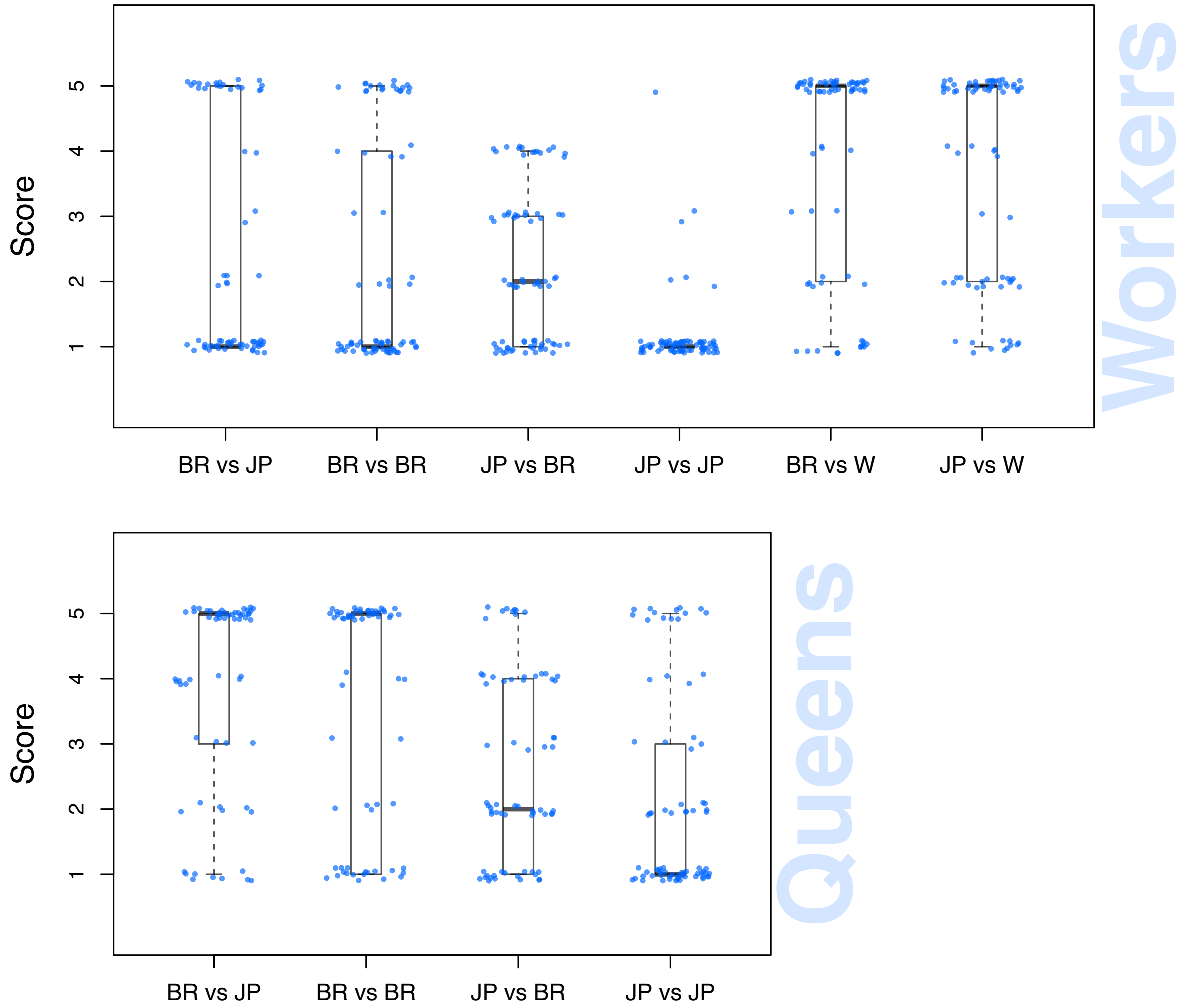

<u>Supplementary Figure 2: Aggression indices in behavioral assay</u>

Workers (top) and queens (bottom) of each lineage (and workers of *W. auropunctata*) were introduced to experimental colonies of either JP or BR. We scored the behavior of the receiving colony based on defined aggression indices and tested for significant differences in potential for high aggressiveness between each of the tested combinations (origin of receiving colony vs. origin of the introduced ant) in a generalized linear model.

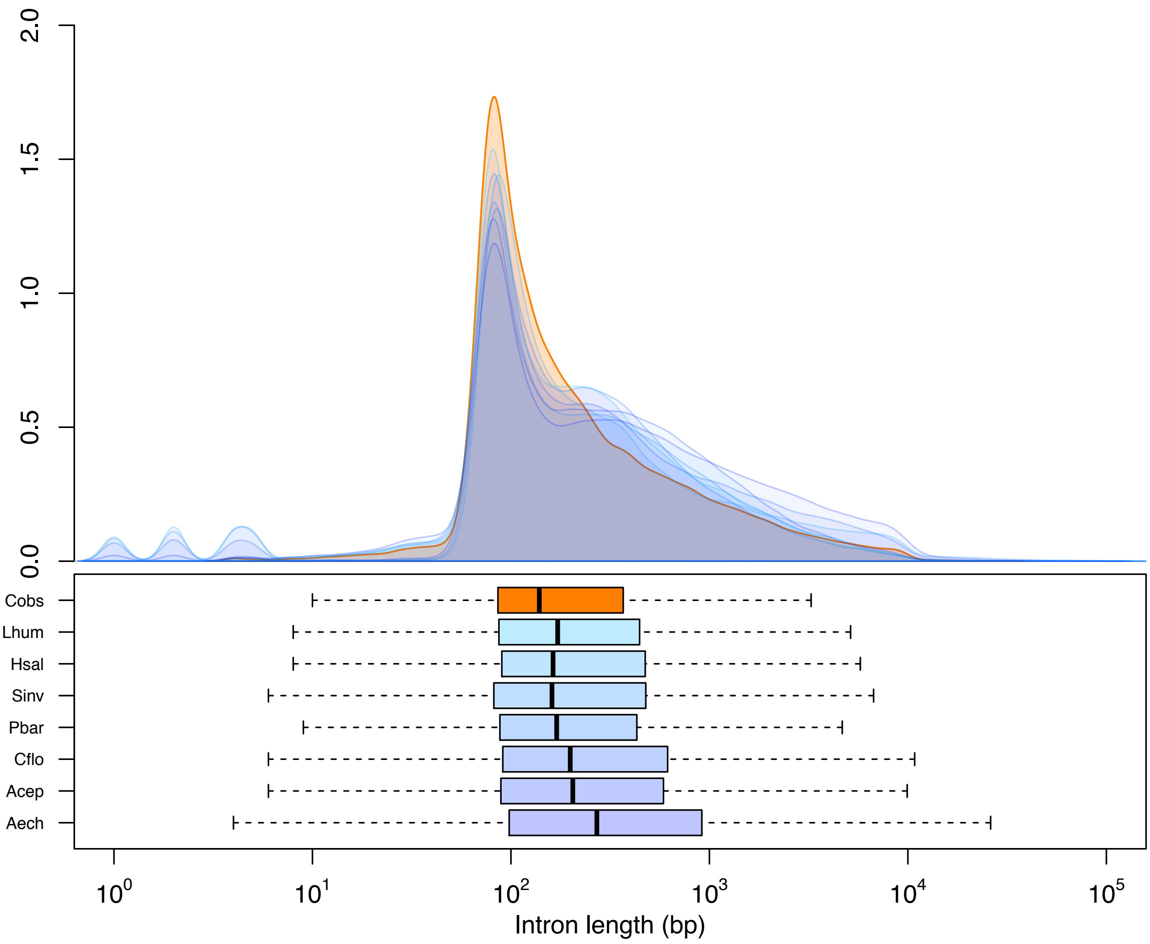

Supplementary Figure 3: Density plot of intron lengths for the sequenced ant genomes

The density plots for intron lengths in *C. obscurior* and seven other published ant genomes show that while the distribution is bimodal in other genomes, the introns of *C. obscurior* deviate from this pattern, with a single peak and a median intron length of 139 bp.

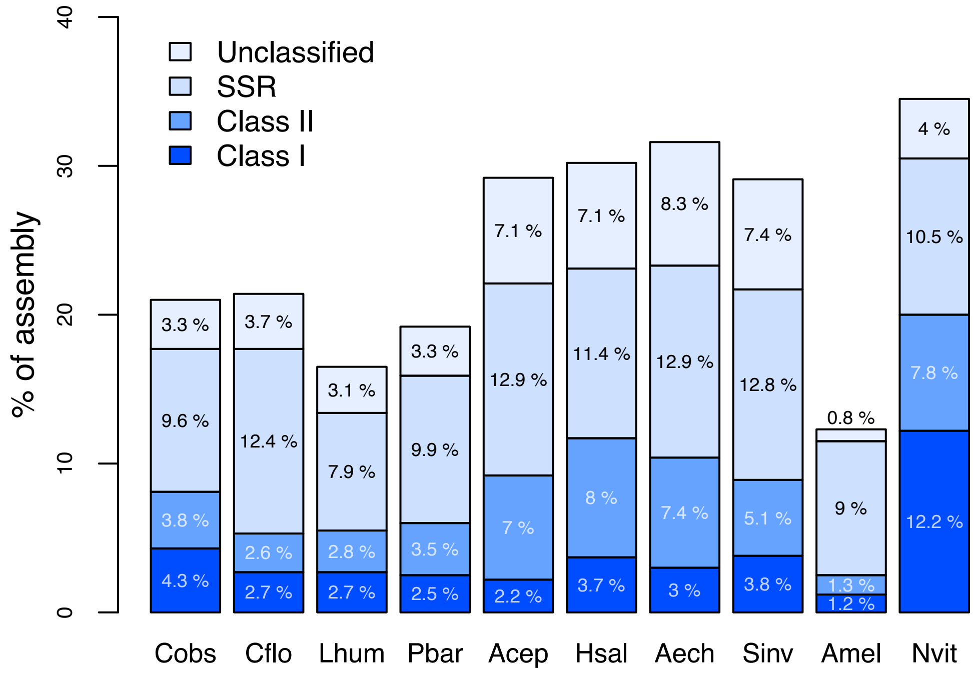

Supplementary Figure 4: Repeat content in sequenced ant genomes relative to assembled genome size

Relative repeat content of *C. obscurior* and nine hymenopteran genomes as calculated from the repeat annotations presented in this study. Across the analyzed ant genomes, repeat content ranges between 16.5 % in *L. humile* to 31.5 % in *A. echinatior*. Relative class I content is higher in *C. obscurior* (4.3 %) than in any of the other ant genomes, yet overall relative repeat content in not different from the smaller genomes (*Cflo*, *Lhum*, *Pbar*). The genomes of *A. mellifera* (*Amel*) and *N. vitripennis* (*Nvit*) are distinct from the analyzed ant genomes in having either much less (*Amel*) or much more (*Nvit*) annotated TEs. SSR = Short simple repeats.

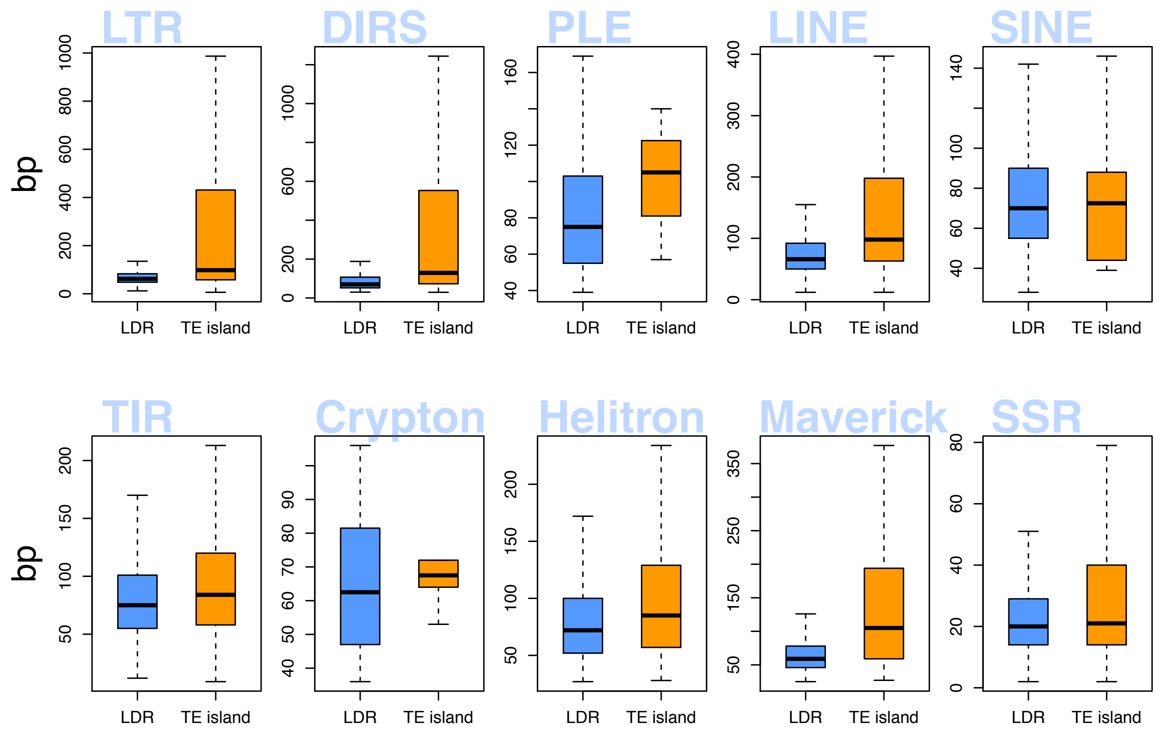

Supplementary Figure 5: Length polymorphism in TE superfamilies and simple repeats between LDRs and TE islands

Length polymorphism in TE superfamilies and simple repeats between LDRs and TE islands. Median element length for all analyzed superfamilies is higher in TE islands than LDRs, suggesting local differences in TE dynamics.

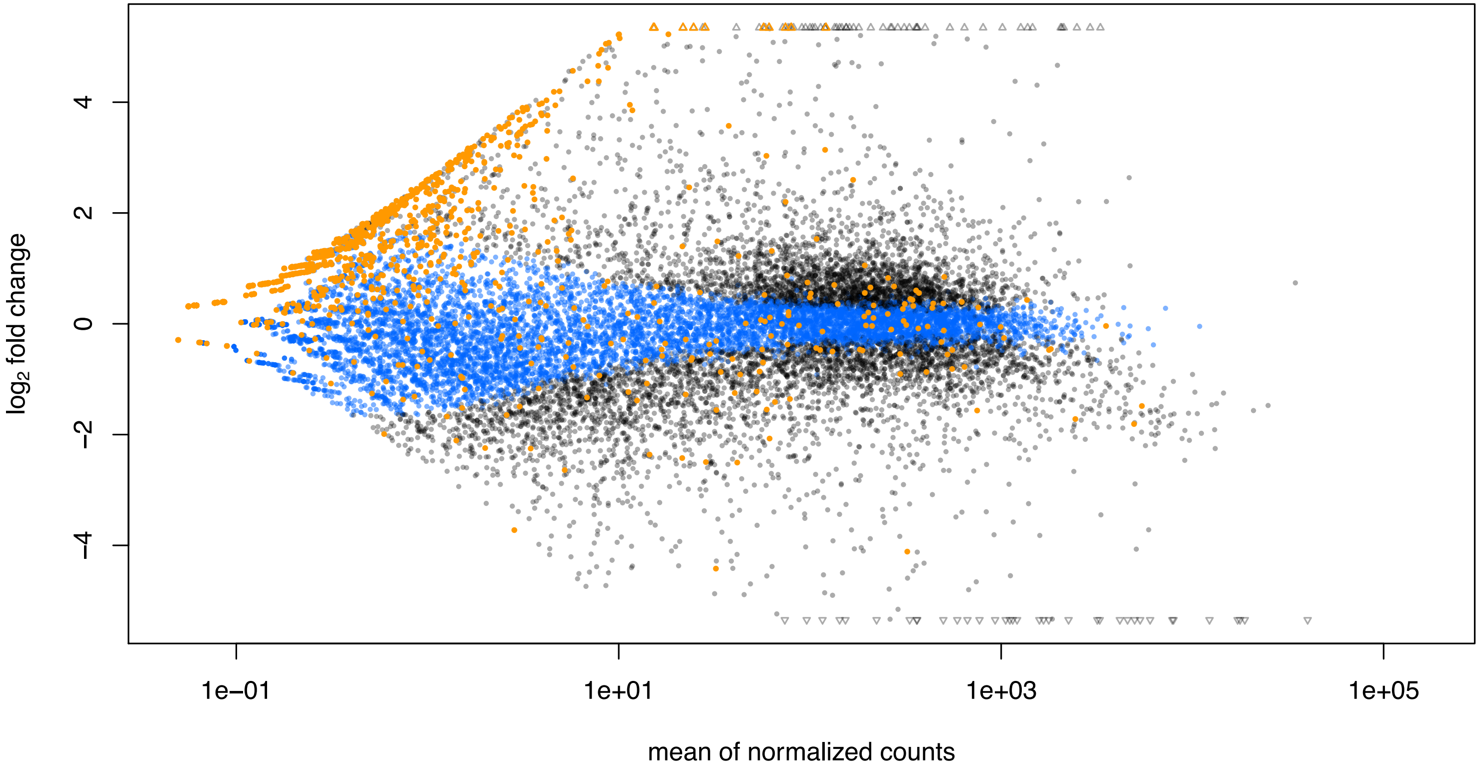

<u>Supplementary Figure 6: MA plot for differential expression of genes between 3rd instar larvae and queens</u>

MA plot for differential expression of genes between 3rd instar larvae and queens. Log ratios of expression of each gene is plotted against the log mean average expression across all samples. Black dots represent genes with significantly different expression. Blue dots show genes that are not significantly different expressed. Genes located in TE islands are plotted in orange. Most TE island genes appear to be more strongly expressed in queens than larvae, while the overall expression of TE island genes is low.

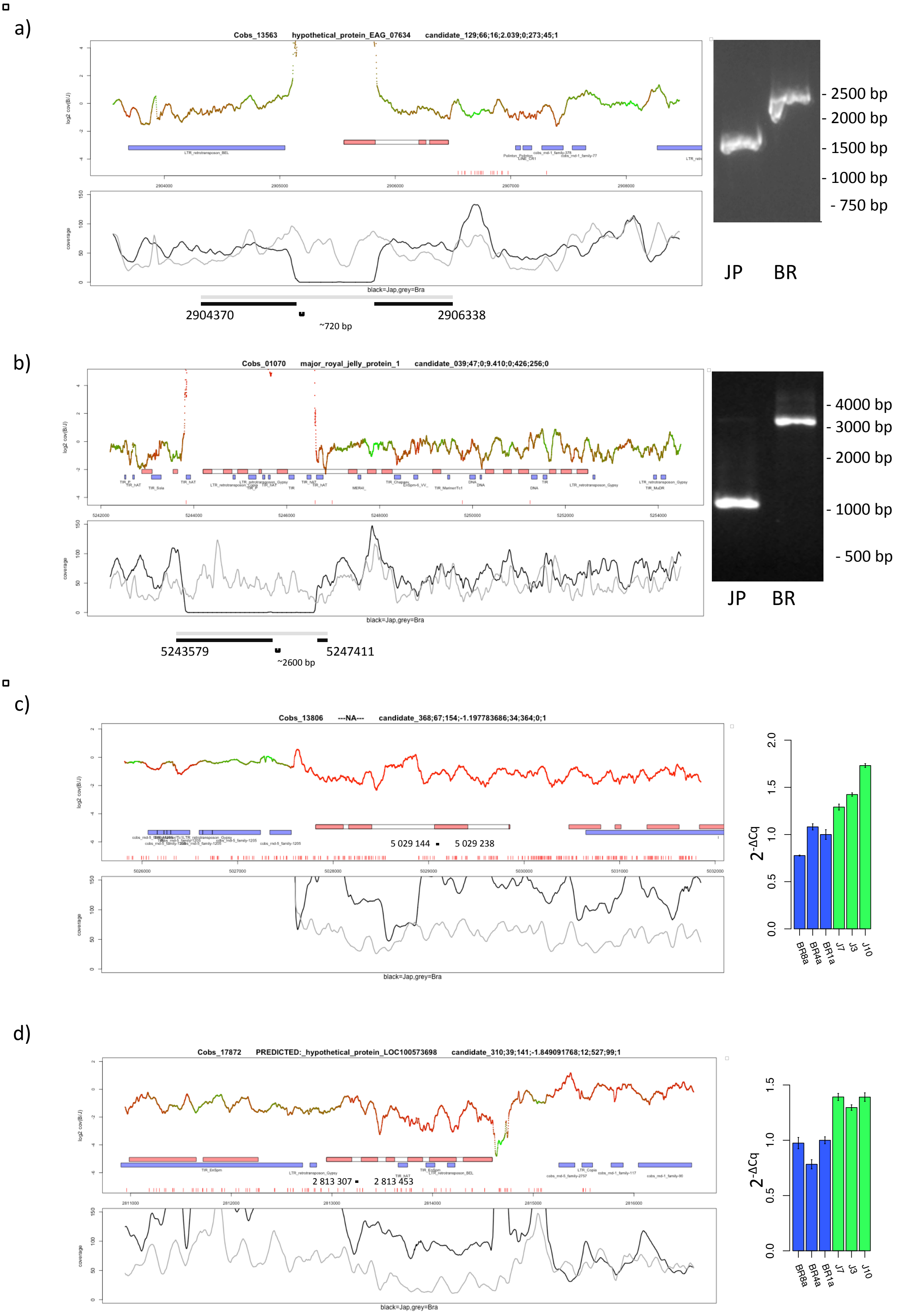

a)
Cobs_13563 hypothetical_protein_EAG_07634 candidate_129;66;16;2.039;0;273;45;1
log2 cov(B/J)
coverage
black=Jap,grey=Bra
2904370
2906338
~720 bp
- 2500 bp
- 2000 bp
- 1500 bp
- 1000 bp
- 750 bp
JP
BR
b)
Cobs_01070 major_royal_jelly_protein_1 candidate_039;47;0;9.410;0;426;256;0
log2 cov(B/J)
coverage
black=Jap,grey=Bra
5243579
5247411
~2600 bp
- 4000 bp
- 3000 bp
- 2000 bp
- 1000 bp
- 500 bp
JP
BR
c)
Cobs_13806 ---NA--- candidate_368;67;154;-1.197783686;34;364;0;1
log2 cov(B/J)
coverage
5 029 144
5 029 238
black=Jap,grey=Bra
2-ΔCq
BR8a
BR4a
BR1a
J7
J3
J10
d)
Cobs_17872 PREDICTED:_hypothetical_protein_LOC100573698 candidate_310;39;141;-1.849091768;12;527;99;1
log2 cov(B/J)
coverage
2 813 307
2 813 453
black=Jap,grey=Bra
2-ΔCq
BR8a
BR4a
BR1a
J7
J3
J10

e)

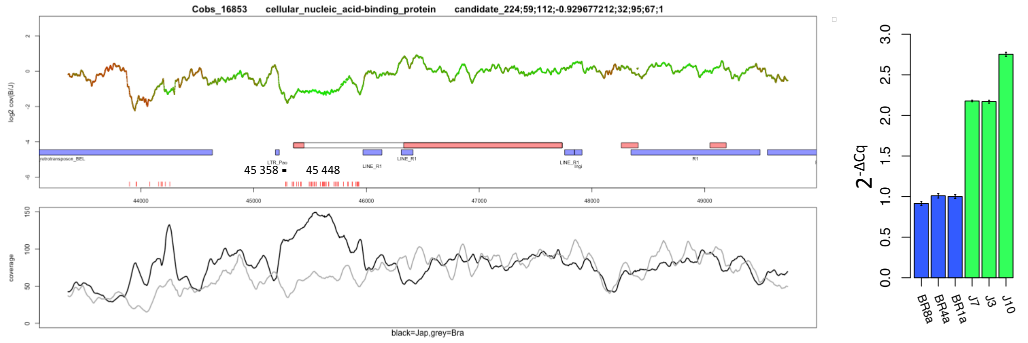

f)

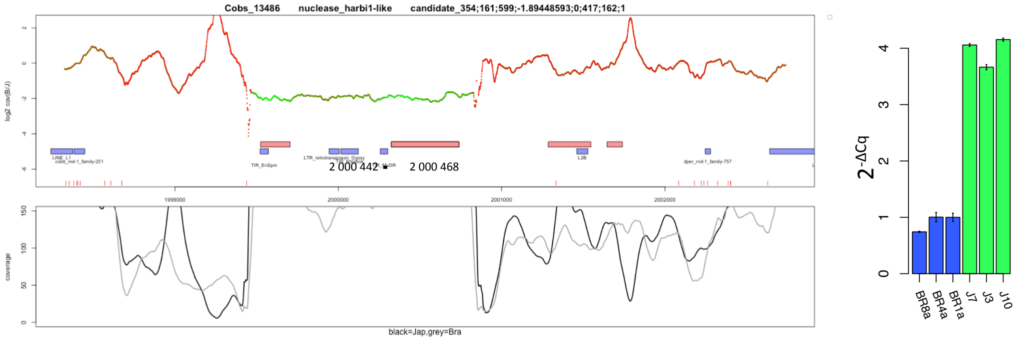

<u>Supplementary Figure 7: Diagnostic plots and experimental confirmation for two deletion candidates (a, b) and four duplication candidates (c-f).</u> Diagnostic plots were created for each of the 512 candidate loci by plotting the log2 JP/BR coverage ratio (red to green), gene models (red boxes), repetitive elements (blue boxes), heterozygous SNV calls (red ticks), and the absolute coverage (grey = BR; black = JP, lower panel). A) Partial deletion of *Cobs_13563* in the JP lineage. PCR and Sanger sequencing confirmed deletion of ~720 bp in the JP genome. B) Deletion of a *MRJP* in *Cobs_01070* in the JP lineage. PCR and Sanger sequencing confirmed deletion of ~2600 bp in the JP genome. C-F) Duplications in *Cobs_13806* (c), *Cobs_17872* (d), *Cobs_16853* (e), and *Cobs_13486* (f) as confirmed by real-time qPCR. $2^{-\Delta Cq}$ values for BR (blue) and JP samples (green) were normalized against colony BR1a. Primer combinations used: *Cobs_13563*: fw: 5'-CAGTTCGGGATGGCGCTC-3', rv: 5'-CGAAAGACTGGGGCTGCAA-3'; *Cobs_01070*: fw: 5'-TCCCGTCAAACCAATCGCAACTCG-3', rv: 5'-TGGGTTGCATCAGGCCACGTA-3'; *Cobs_13806*: fw: 5'-GCAACGGTGCTCACAGGAGCC-3', rv: 5'-AAAGGCGATGCCCTCCGTTGC-3'; *Cobs_17872*: fw: 5'-TCGTAGACGATTATATAGAGCG-3', rv: 5'-GTAGCAGAAGTAGAAGGCATTGG-3'; *Cobs_13486*: fw: 5'-TCATTGACATCGAATTCGTCATGGCTG-3', rv: 5'-AACGTGTAATGGCTGCTGCTATACTTC-3'; *Cobs_16853*: fw: 5'-GCGACGTCGAGATAAAGGTTTCG-3', rv: 5'-CGTTAATTGGTAGGGTTCGC-3'.

Supplementary Tables

Supplementary Table 1: 22 compounds in cuticle extracts of BR and JP used for statistics

ID	Compound
**1**	x-Hexadecene
**2**	Hexadecane (C16)
**3**	Heptadecane (C17)
**4**	Octadecane (C18)
**5**	Nonadecane (C19)
**6**	Pentacosane (C25)
**7**	3-methyl pentacosane
**8**	x-Heptacosene
**9**	Heptacosane (C27)
**10**	3-methyl heptacosane
**11**	x-methyl octacosane
**12**	x-nonacosene
**13**	Nonacosane (C29)
**14**	13-methyl and 15-methyl nonacosane
**15**	Hentriacontane (C31)
**16**	13-methyl hentriacontane
**17**	11-methyl tritriacontane
**18**	x,x-dimethyl tritriacontane
**19**	x-methyl pentatriacontane
**20**	x,x-dimethyl pentatriacontane (prop. 13,23)
**21**	x-methyl heptatriacontane (prop. 13)
**22**	x,x-dimethyl heptatriacontane

Supplementary Table 2: Data used for the *C. obscurior* draft genome assembly. Genome coverage computed assuming 195 Mb estimated genome size

<table>
<tr><th></th><th>No. of reads</th><th>Average length (bp)</th><th>Genome coverage</th></tr>
<tr><td>220 bp Illumina paired end</td><td>209 740 014</td><td>100</td><td>105x</td></tr>
<tr><td>8 Kb paired end (reads)</td><td>1 318 264</td><td rowspan="2">189</td><td rowspan="2">1.2x</td></tr>
<tr><td>8 Kb paired end (valid pairs)</td><td>416 174</td></tr>
<tr><td>20 Kb paired end (reads)</td><td>1 131 046</td><td rowspan="2">194</td><td rowspan="2">1.1x</td></tr>
<tr><td>20 Kb paired end (valid pairs)</td><td>326 815</td></tr>
</table>

Supplementary Table 3: Quantitative assembly statistics for Cobs1.4

**Scaffolded sequence (bp)**	177 892 999
**N50 scaffold size (bp)**	3 105 814
**Total number of scaffolds**	1 854
**GC content**	0.3958
**Total no. of annotated genes**	17 552
**Total no. of gene models with AED<0.5**	12 752
**Total no. of genes with Interpro domain**	9 552
**Scaffolded sequence (bp)**	177 892 999

Supplementary Table 4: Comparison of gene body, exon and intron structure of *C. obscurior* and other analyzed ant genomes

	Gene Body				Exon				Intron			
**Species**	**Median length (bp)**	**Total length (Mbp)**	**Count**	**% of assembly**	**Median length (bp)**	**Total length (Mbp)**	**Count**	**% of assembly**	**Median length (bp)**	**Total length (Mbp)**	**Count**	**% of assembly**
C. floridanus	1790	71.85	17059	29.94%	179	20.75	83401	8.64%	199	51.10	66342	21.29%
P. barbatus	1838	52.51	17152	22.34%	180	20.49	81657	8.72%	170	32.03	64504	13.63%
H. saltator	1273	72.33	18561	24.35%	184	20.41	78601	6.87%	163	51.91	60040	17.48%
L. humile	2068	54.00	16097	25.04%	181	20.48	80622	9.50%	172	33.52	64525	15.55%
S. invicta	1365	44.30	16522	12.55%	182	17.25	66992	4.89%	161	27.06	50470	7.66%
A. echinatior	2783	92.35	17278	30.78%	180	21.00	85030	7.00%	271	71.35	67752	23.78%
A. cephalotes	1932	59.72	18090	18.84%	169	19.53	83900	6.16%	205	40.19	65810	12.68%
C. obscurior	1844	60.35	17552	33.92%	171	22.14	92173	12.45%	139	38.21	74621	21.48%

Supplementary Table 5: Relative content (%) of repetitive elements and TEs in the genomes of *C. obscurior* and other Hymenoptera

Type	Repeat	Cobs	Cflo	Lhum	Pbar	Acep	Hsal	Aech	Sinv	Amel	Nvit
LTR	Gypsy	**1.643**	0.94	1	1.247	0.725	1.284	0.901	1.216	0.389	4.754
LTR	Copia	0.369	0.227	0.247	0.179	0.147	0.348	0.2	0.4	0.131	0.957
LTR	BEL	**0.499**	0.244	0.264	0.151	0.158	0.206	0.266	0.485	0.061	0.488
LTR	DIRS	**0.095**	0.008	0.072	0.027	0.018	0.065	0.019	0.072	0.003	0.089
LTR	Ngaro	**0.018**	0	0	0	0	0	0	0	0	0
LTR	Pao	0.033	0.034	0.099	0.024	0.083	0.023	0.206	0.02	0.043	0.167
LTR	ERV1	0.081	0.056	0.021	0.061	0.079	0.093	0.09	0.074	0.045	0.055
LTR	ERV2	**0.041**	0.027	0.01	0.025	0.019	0.037	0.022	0.02	0.024	0.027
LTR	ERV3	0.01	0.007	0.002	0.007	0.006	0.01	0.007	0.006	0.006	0.008
LTR	ERVK	0	0	0	0	0	0	0	0	0	0.146
LTR	ERVL	0	0.001	0.001	0	0	0.004	0	0	0	0
LTR	Unclassified	0.307	0.392	0.152	0.089	0.162	0.202	0.257	0.358	0.142	1.558
LINE	CR1	**0.232**	0.091	0.035	0.061	0.059	0.156	0.063	0.121	0.073	1.349
LINE	L1	**0.163**	0.071	0.022	0.063	0.057	0.136	0.065	0.057	0.067	0.113
LINE	L2	0.034	0.025	0.034	0.035	0.113	0.025	0.196	0.061	0.007	0.093
LINE	L2A	**0.002**	0	0	0	0	0.001	0	0	0	0.001
LINE	L2B	0.007	0.004	0.002	0.021	0.002	0.009	0.003	0.009	0.001	0.004
LINE	Jockey	0.033	0.038	0.022	0.02	0.016	0.04	0.02	0.023	0.01	0.034
LINE	LOA	0.046	0.006	0.088	0.017	0.007	0.01	0.007	0.068	0.003	0.303
LINE	R1	0.199	0.144	0.255	0.119	0.065	0.667	0.136	0.315	0.02	0.253
LINE	R2	**0.032**	0.008	0.014	0.006	0.003	0.023	0.004	0.014	0.007	0.034
LINE	R4	0.015	0.008	0.008	0.008	0.007	0.009	0.007	0.007	0.012	0.012
LINE	RTEX	**0.007**	0.004	0.003	0.005	0.003	0.003	0.002	0.006	0.001	0.01
LINE	Penelope	0.04	0.071	0.068	0.097	0.221	0.035	0.269	0.12	0.01	0.113
LINE	RTE	0.149	0.088	0.146	0.087	0.071	0.147	0.083	0.182	0.047	0.243
LINE	CRE	**0.005**	0.002	0	0.002	0.001	0.003	0.001	0.001	0.001	0.003
LINE	NeSL	**0.033**	0.011	0.005	0.009	0.009	0.01	0.006	0.004	0.009	0.016
LINE	Rex1	0.002	0.003	0.003	0.002	0.001	0.009	0.001	0.025	0.008	0.002
LINE	Randl	0.001	0.001	0.001	0.001	0.001	0.001	0.001	0.001	0.001	0.002
LINE	Tx1	**0.019**	0.007	0.001	0.007	0.006	0.014	0.007	0.005	0.006	0.012
LINE	Crack	**0.035**	0.006	0.001	0.005	0.007	0.021	0.007	0.006	0.006	0.018
LINE	Nimb	0.006	0.004	0.002	0.003	0.002	0.011	0.004	0.008	0.003	0.008
LINE	Proto1	0.005	0.001	0	0.002	0.002	0.005	0.001	0.001	0.002	0.004
LINE	Proto2	**0.003**	0.001	0	0.001	0.001	0.002	0.001	0.001	0.001	0.002
LINE	Hero	0	0	0	0	0	0	0	0	0	0
LINE	Tad1	0	0	0	0	0	0	0	0.003	0	0
LINE	Ingi	0.001	0.001	0.002	0.001	0	0.002	0.001	0.001	0.001	0.003
LINE	Outcast	**0.007**	0.002	0	0.001	0.002	0.004	0.003	0.002	0.004	0.006
LINE	Daphne	**0.002**	0.001	0	0	0	0.001	0	0.001	0	0.001
LINE	Ambal	0.001	0.001	0	0.001	0.001	0.001	0.001	0	0.001	0
LINE	Vingi	0.001	0	0	0.001	0.001	0.001	0	0	0	0.001
LINE	I	**0.05**	0.047	0.039	0.023	0.027	0.022	0.025	0.049	0.008	1.136
LINE	DRE	0.001	0.001	0.002	0	0	0	0	0	0.001	0.002

**LINE**	**telomeric**	0.007	0.004	0.012	0.004	0.003	0.013	0.003	0.003	0.002	0.016
**LINE**	**Unclassified**	**0.015**	0.006	0.006	0.005	0.008	0.013	0.006	0.005	0.006	0.038
**SINE**	**SINE1_7SL**	0	0	0	0	0	0	0	0	0	0
**SINE**	**SINE2_trna**	0.012	0.058	0.004	0.009	0.003	0.006	0.004	0.003	0.002	0.014
**SINE**	**SINE3_5S**	0.001	0.001	0.001	0.002	0.001	0.037	0.001	0.002	0.002	0.002
**SINE**	**SINE_MIR**	0	0.035	0.064	0.012	0.006	0.001	0.005	0	0.001	0
**SINE**	**SINE_B4**	0	0	0	0	0	0	0	0	0	0.006
**SINE**	**SINE_RTE**	0	0	0	0.001	0	0	0	0	0	0
**SINE**	**SINE_L1**	0	0	0	0	0	0.001	0	0.001	0	0
**SINE**	**SINE_R1**	0	0	0	0	0	0	0	0	0	0.013
**SINE**	**Unclassified**	0.023	0.017	0.006	0.027	0.048	0.022	0.05	0.018	0.009	0.024
**Unclassified classI**		**0.02**	0.008	0.002	0.007	0.01	0.016	0.012	0.01	0.007	0.02
**TIR**	**hAT**	**0.554**	0.219	0.188	0.251	0.403	0.501	0.447	0.399	0.152	0.392
**TIR**	**Mariner**	0.346	0.285	0.255	0.561	1.527	2.283	1.454	0.623	0.297	0.279
**TIR**	**MuDR**	**0.262**	0.072	0.027	0.053	0.06	0.167	0.059	0.05	0.071	0.157
**TIR**	**EnSpm**	**0.531**	0.256	0.277	0.302	0.236	0.487	0.253	0.324	0.153	0.65
**TIR**	**piggyBac**	0.032	0.014	0.009	0.006	0.01	0.042	0.01	0.017	0.023	0.018
**TIR**	**P**	**0.123**	0.049	0.071	0.021	0.025	0.08	0.031	0.063	0.02	0.076
**TIR**	**Merlin**	0.01	0.003	0.002	0.008	0.013	0.012	0.014	0.006	0.002	0.003
**TIR**	**Harbinger**	**0.073**	0.028	0.013	0.021	0.019	0.05	0.02	0.021	0.022	0.047
**TIR**	**Transib**	0.063	0.028	0.037	0.015	0.019	0.174	0.02	0.035	0.015	0.09
**TIR**	**Novosib**	0.002	0.001	0.001	0.001	0.001	0.009	0.001	0.001	0.001	0.007
**TIR**	**Mirage**	0.001	0.001	0	0	0.001	0.001	0	0	0	0.001
**TIR**	**Rehavkus**	**0.041**	0.028	0.02	0.009	0.007	0.024	0.009	0.034	0.005	0.084
**TIR**	**Kolobok**	0.058	0.031	0.034	0.019	0.023	0.045	0.034	0.078	0.006	0.278
**TIR**	**ISL2EU**	**0.011**	0.002	0.001	0.001	0.002	0.005	0.001	0.002	0.002	0.008
**TIR**	**Chapaev**	0.072	0.08	0.031	0.089	0.137	0.253	0.18	0.144	0.02	0.126
**TIR**	**Crypton**	**0.002**	0.001	0	0	0	0.001	0.001	0.001	0	0.035
**TIR**	**Sola**	0.105	0.117	0.101	0.034	0.083	0.788	0.099	0.187	0.027	0.212
**TIR**	**Zator**	0.009	0.002	0	0.002	0.002	0.005	0.002	0.003	0.002	0.006
**TIR**	**Ginger1**	0.039	0.015	0.004	0.007	0.01	0.035	0.011	0.007	0.009	0.026
**TIR**	**Ginger2/TDD**	**0.022**	0.004	0.001	0.003	0.003	0.009	0.004	0.004	0.005	0.01
**TIR**	**Academ**	0.011	0.008	0.026	0.001	0.004	0.008	0.004	0.018	0.002	0.024
**TIR**	**Other TIR**	0.867	1.001	1.369	1.168	3.764	2.283	4.034	2.808	0.359	0.824
**MITE**	**MITE**	0	0	0	0	0	0	0	0	0	0
**Helitron**	**Helitron**	**0.235**	0.099	0.078	0.127	0.08	0.181	0.09	0.071	0.07	2.938
**Polinton**	**Polinton**	0.325	0.158	0.224	0.822	0.509	0.408	0.543	0.226	0.051	1.452
**Unclassified classII**		0.005	0.11	0.036	0.012	0.017	0.103	0.032	0.019	0.006	0.042
**SSR**	**Simple repeat**	0.419	0.734	0.519	1.072	1.205	0.851	1.409	0.794	0.379	0.41
**SSR**	**Low complexity**	0.861	3.196	3.438	2.935	5.925	2.806	6.629	6.662	1.288	2.225
**SSR**	**Satellite**	0.01	0.042	0.019	0.007	0.006	0.011	0.006	0.015	0.004	1.747
**SSR**	**Other**	0.01	0.042	0.019	0.007	0.006	0.011	0.006	0.015	0.004	1.747
**SSR**	**Unclassified**	8.325	8.394	3.933	5.884	5.795	7.73	4.838	5.338	7.355	4.376
**Unclassified**		3.257	3.75	3.091	3.32	7.14	7.107	8.254	7.398	0.76	3.996

Supplementary Table 6: Enrichment of TE superfamilies in TE islands

Element type	Total bp in TE islands	Total bp in LDRs	Total number in TE islands	Total number in LDRs	FDR (base count)	FDR (element number)
Unclassified	1892769	4847567	9616	41643	>4.53E-155	>4.53E-155
TcMar-Tc1	84041	22152	515	164	>4.53E-155	>4.53E-155
DIRS	125721	30503	330	201	>4.53E-155	4.53E-155
RTE	138967	64262	309	502	>4.53E-155	5.68E-73
Ngaro	22579	5560	115	31	>4.53E-155	1.99E-72
TcMar-Mariner	9797	686	76	9	>4.53E-155	2.05E-56
Maverick	51886	64613	353	865	>4.53E-155	1.14E-49
LOA	18863	3596	67	53	>4.53E-155	4.80E-28
Kolobok-Hydra	6187	1190	20	7	>4.53E-155	4.24E-12
on	2804	1230	19	11	>4.53E-155	1.10E-09
Loa	38653	13148	42	93	>4.53E-155	1.57E-07
R1	121215	152620	286	1357	>4.53E-155	3.05E-07
BEL	553183	285447	560	2984	>4.53E-155	5.48E-07
Academ	5341	13540	52	167	0.033	3.75E-05
Merlin	6610	8835	41	120	>4.53E-155	5.32E-05
R2	20282	31169	37	139	>4.53E-155	0.007

Supplementary Table 7: List of duplication/deletion candidate loci and intersection with Cobs1.4 annotated genes

DUPLICATIONS										
**Scf**	**Start**	**Stop**	**Name;covBR;covJP;log2ratio;JPhet;exon bases;TE bases; island_binary**	**Het SNV**	**In**	**Affected gene**	**Type**	**Gene alias**	**Isl.**	**RNA seq**
scf0022	552001	553001	candidate_002;191;109;0.807;2;781;99;0	2	BR	Cobs_06524	Single exon duplication	upf0468_protein_cg5343-like	NO	**YES**
scf0022	552001	553001	candidate_002;191;109;0.807;2;781;99;0	2	BR	Cobs_06530	Single exon duplication	transcription_initiation_factor_tfiid_subunit_9-like	NO	**YES**
scf0072	9001	10001	candidate_004;121;64;0.922;0;1000;115;0	0	BR	Cobs_15626	Partial exon duplication	pin2-interacting_protein_x1	NO	**YES**
scf0028	384001	385001	candidate_007;65;130;-1.002546172;4;215;102;0	4	JP	Cobs_04037	Whole gene duplication		NO	**YES**
scf0030	1215001	1216001	candidate_007;96;54;0.820;4;373;193;0	4	BR	Cobs_05921	Multiple exon duplication	odorant_receptor_168	NO	**YES**
scf0055	661001	662001	candidate_014;81;268;-1.732135971;1;459;52;0	1	JP	Cobs_10648	Multiple exon duplication	hypothetical_protein_G5I_05212	NO	**YES**
scf0044	556001	557001	candidate_016;441;160;1.467;8;1000;0;0	8	BR	Cobs_02801	Partial exon duplication	Mucin-1	NO	**YES**
scf0010	108001	109001	candidate_037;368;128;1.522;9;273;214;0	9	BR	Cobs_00609	Multiple exon duplication		NO	NO
scf0003	617001	618001	candidate_045;175;94;0.900;0;128;0;1	0	BR	Cobs_14275	Multiple exon duplication		**YES**	NO
scf0003	1714001	1715001	candidate_046;139;78;0.839;6;903;0;0	6	BR	Cobs_14337	Whole gene duplication	85_kda_calcium-independent_phospholipase_a2-like	NO	**YES**
scf0003	1715001	1716001	candidate_047;228;98;1.210;10;1000;0;0	10	BR	Cobs_14337	Whole gene duplication	85_kda_calcium-independent_phospholipase_a2-like	NO	**YES**
scf0003	1716001	1717001	candidate_048;128;58;1.129;8;455;0;0	8	BR	Cobs_14337	Whole gene duplication	85_kda_calcium-independent_phospholipase_a2-like	NO	**YES**
scf0002	1474001	1475001	candidate_059;323;78;2.054;32;973;24;1	32	BR	Cobs_17748	Whole gene duplication		**YES**	**YES**
scf0002	2683001	2684001	candidate_075;99;54;0.887;7;347;346;1	7	BR	Cobs_17834	Multiple exon duplication	cytochrome_p450_4c1	**YES**	**YES**
scf0035	1363001	1364001	candidate_092;52;102;-0.95364953;14;406;343;0	14	JP	Cobs_04670	Multiple exon duplication	coiled-coil_domain-containing_protein_95	NO	**YES**
scf0035	1364001	1365001	candidate_093;66;132;-1.009649218;25;780;0;0	25	JP	Cobs_04689	Multiple exon duplication	sin3_histone_deacetylase_corepressor_complex_component_sds3-like	NO	**YES**
scf0001	4465001	4466001	candidate_094;80;20;2.000;5;313;243;1	5	BR	Cobs_07170	Multiple exon duplication	hypothetical_protein_EAI_00174	**YES**	**YES**
scf0001	4466001	4467001	candidate_095;148;48;1.628;4;628;59;1	4	BR	Cobs_07170	Multiple exon duplication	hypothetical_protein_EAI_00174	**YES**	**YES**
scf0042	2001	3001	candidate_098;119;217;-0.873101452;4;349;329;0	4	JP	Cobs_15501	Multiple exon duplication	nucleoside_diphosphate_kinase_7	NO	**YES**
scf0001	4823001	4824001	candidate_101;371;176;1.075;10;647;7;1	10	BR	Cobs_07201	Multiple exon duplication	hypothetical_protein_SINV_05299	**YES**	**YES**

scf0007	1468001	1469001	candidate_115;216;107;1.021;0;126;0;1	0	BR	Cobs_13418	Multiple exon duplication	odorant_receptor_13a	**YES**	NO
scf0007	1469001	1470001	candidate_116;213;99;1.107;0;366;0;1	0	BR	Cobs_13416	Whole gene duplication		**YES**	NO
scf0037	73001	74001	candidate_123;89;156;-0.812559297;10;129;0;0	10	JP	Cobs_00477	Whole gene duplication		NO	**YES**
scf0037	122001	123001	candidate_127;61;157;-1.372576506;6;452;153;0	6	JP	Cobs_00487	Whole gene duplication	nicotinic_acetylcholine_receptor_subunit_alpha_6_ transcript_variant_partial	NO	**YES**
scf0037	122001	123001	candidate_127;61;157;-1.372576506;6;452;153;0	6	JP	Cobs_00483	Whole gene duplication		NO	NO
scf0037	126001	127001	candidate_128;59;150;-1.354662195;5;310;0;0	5	JP	Cobs_00482	Whole gene duplication		NO	**YES**
scf0037	952001	953001	candidate_129;49;101;-1.056522402;8;185;126;0	8	JP	Cobs_00543	Whole gene duplication		NO	NO
scf0037	953001	954001	candidate_130;53;100;-0.900691163;5;366;90;0	5	JP	Cobs_00543	Whole gene duplication		NO	NO
scf0018	2159001	2160001	candidate_143;58;104;-0.846853003;15;743;0;0	15	JP	Cobs_09630	Multiple exon duplication	hypothetical_protein_EAG_01487	NO	**YES**
scf0025	148001	149001	candidate_149;33;89;-1.441702554;20;274;165;0	20	JP	Cobs_06204	Whole gene duplication	hypothetical_protein_SINV_03739	NO	NO
scf0012	754001	755001	candidate_178;67;128;-0.935076915;11;535;291;1	11	JP	Cobs_15866	Multiple exon duplication	fatty_acid_synthase	**YES**	NO
scf0004	4177001	4178001	candidate_215;44;78;-0.840153732;25;533;0;0	25	JP	Cobs_05107	Whole gene duplication	isoform_a	NO	**YES**
scf0004	4177001	4178001	candidate_215;44;78;-0.840153732;25;533;0;0	25	JP	Cobs_05102	Whole gene duplication		NO	NO
scf0004	4178001	4179001	candidate_216;58;107;-0.890949904;31;791;0;0	31	JP	Cobs_05107	Whole gene duplication	isoform_a	NO	**YES**
scf0004	4178001	4179001	candidate_216;58;107;-0.890949904;31;791;0;0	31	JP	Cobs_05102	Whole gene duplication		NO	NO
scf0004	4179001	4180001	candidate_217;46;128;-1.456836393;38;848;78;0	38	JP	Cobs_05107	Whole gene duplication	isoform_a	NO	**YES**
scf0004	4179001	4180001	candidate_217;46;128;-1.456836393;38;848;78;0	38	JP	Cobs_05102	Whole gene duplication		NO	NO
scf0004	4180001	4181001	candidate_218;90;173;-0.952840484;34;1000;0;0	34	JP	Cobs_05107	Whole gene duplication	isoform_a	NO	**YES**
scf0004	4180001	4181001	candidate_218;90;173;-0.952840484;34;1000;0;0	34	JP	Cobs_05102	Whole gene duplication		NO	NO
scf0004	5302001	5303001	candidate_219;39;74;-0.936086412;0;542;200;0	0	JP	Cobs_05246	Whole gene duplication	iron-sulfur_cluster_co-chaperone_protein_mitochondrial-like	NO	**YES**
scf0009	45001	46001	candidate_224;59;112;-0.929677212;32;95;67;1	32	JP	Cobs_16853	Single exon duplication	cellular_nucleic_acid-binding_protein	**YES**	NO
scf0009	289001	290001	candidate_226;50;109;-1.114057336;12;395;50;1	12	JP	Cobs_16890	Whole gene duplication	adenylate_cyclase_type_10	**YES**	NO
scf0009	580001	581001	candidate_229;70;182;-1.371314387;33;729;0;1	33	JP	Cobs_16903	Whole gene duplication	hypothetical_protein_EAG_04423	**YES**	NO
scf0009	4113001	4114001	candidate_238;22;50;-1.194886622;0;185;141;0	0	JP	Cobs_17195	Whole gene duplication		NO	**YES**
scf0009	5349001	5350001	candidate_241;48;92;-0.942831796;10;529;251;1	10	JP	Cobs_17316	Whole gene duplication		**YES**	NO
scf0009	5638001	5639001	candidate_243;53;112;-1.081607453;9;821;410;1	9	JP	Cobs_17356	Whole gene duplication	PREDICTED:_uncharacterized_protein_K02A2.6-like	**YES**	NO
scf0010	155001	156001	candidate_252;43;115;-1.419704346;4;561;463;0	4	JP	Cobs_00607	Multiple exon duplication	hypothetical_protein_EAG_02592	NO	NO

scf0010	5129001	5130001	candidate_259;29;64;-1.157054559;0;153;34;0	0	JP	Cobs_01066	Whole gene duplication		NO	NO
scf0006	5610001	5611001	candidate_267;38;75;-0.971717664;0;106;56;0	0	JP	Cobs_09179	Multiple exon duplication		NO	NO
scf0008	2911001	2912001	candidate_276;46;102;-1.137478081;1;314;57;1	1	JP	Cobs_16467	Whole gene duplication		**YES**	NO
scf0008	3313001	3314001	candidate_278;33;66;-1.002599274;0;458;0;0	0	JP	Cobs_16545	Whole gene duplication	hypothetical_protein_SINV_06963	NO	**YES**
scf0008	3313001	3314001	candidate_278;33;66;-1.002599274;0;458;0;0	0	JP	Cobs_16554	Whole gene duplication		NO	NO
scf0003	85001	86001	candidate_282;47;81;-0.802449243;12;618;0;1	12	JP	Cobs_14245	Partial exon duplication	jerky_protein_homolog-like	**YES**	NO
scf0003	480001	481001	candidate_283;25;48;-0.923793684;0;207;151;1	0	JP	Cobs_14265	Multiple exon duplication	odorant_receptor_13a	**YES**	NO
scf0003	712001	713001	candidate_285;121;269;-1.155246806;8;502;42;1	8	JP	Cobs_14284	Multiple exon duplication	hypothetical_protein_G5I_09119	**YES**	NO
scf0003	943001	944001	candidate_286;35;63;-0.838878536;0;323;168;0	0	JP	Cobs_14297	Multiple exon duplication	transcriptional_regulator_atrx	NO	**YES**
scf0003	3184001	3185001	candidate_292;28;83;-1.546172902;9;365;116;1	9	JP	Cobs_14441	Whole gene duplication	hypothetical_protein_SINV_08869	**YES**	**YES**
scf0002	1440001	1441001	candidate_301;50;153;-1.605684692;0;448;174;1	0	JP	Cobs_17749	Whole gene duplication		**YES**	NO
scf0002	1441001	1442001	candidate_302;12;29;-1.293020781;0;212;113;1	0	JP	Cobs_17749	Whole gene duplication		**YES**	NO
scf0002	1723001	1724001	candidate_305;46;89;-0.937334261;7;177;120;1	7	JP	Cobs_17787	Multiple exon duplication		**YES**	NO
scf0002	2337001	2338001	candidate_307;88;346;-1.980760634;18;433;245;1	18	JP	Cobs_17885	Whole gene duplication	tyrosine_recombinase	**YES**	NO
scf0002	2813001	2814001	candidate_309;41;114;-1.470732135;11;632;163;1	11	JP	Cobs_17872	Whole gene duplication	PREDICTED:_hypothetical_protein_LOC100573698	**YES**	NO
scf0002	2814001	2815001	candidate_310;39;141;-1.849091768;12;527;99;1	12	JP	Cobs_17872	Whole gene duplication	PREDICTED:_hypothetical_protein_LOC100573698	**YES**	NO
scf0002	3224001	3225001	candidate_312;47;130;-1.477483194;4;871;0;1	4	JP	Cobs_17892	Whole gene duplication	gustatory_receptor_28b	**YES**	NO
scf0002	3432001	3433001	candidate_313;56;113;-1.01704099;32;462;0;1	32	JP	Cobs_17925	Multiple exon duplication	hypothetical_protein_EAG_00455	**YES**	NO
scf0002	3432001	3433001	candidate_313;56;113;-1.01704099;32;462;0;1	32	JP	Cobs_17927	Multiple exon duplication	hypothetical_protein_EAG_00088	**YES**	NO
scf0001	2003001	2004001	candidate_322;67;132;-0.96762421;11;791;0;1	11	JP	Cobs_07015	Whole gene duplication	serine_threonine-protein_kinase	**YES**	NO
scf0001	2003001	2004001	candidate_322;67;132;-0.96762421;11;791;0;1	11	JP	Cobs_07016	Whole gene duplication		**YES**	NO
scf0007	1234001	1235001	candidate_350;255;456;-0.839904468;9;279;0;1	9	JP	Cobs_13394	Whole gene duplication		**YES**	NO
scf0007	1463001	1464001	candidate_352;34;60;-0.833221343;0;340;127;1	0	JP	Cobs_13418	Multiple exon duplication	odorant_receptor_13a	**YES**	NO
scf0007	1999001	2000001	candidate_353;118;396;-1.750453075;0;182;109;1	0	JP	Cobs_13483	Multiple exon duplication	uncharacterized_aminotransferase_sso0104	**YES**	**YES**
scf0007	2000001	2001001	candidate_354;161;599;-1.89448593;0;417;162;1	0	JP	Cobs_13483	Multiple exon duplication	uncharacterized_aminotransferase_sso0104	**YES**	**YES**
scf0007	2000001	2001001	candidate_354;161;599;-1.89448593;0;417;162;1	0	JP	Cobs_13486	Whole gene duplication	nuclease_harbi1-like	**YES**	NO
scf0007	4178001	4179001	candidate_361;66;122;-0.899327359;6;709;183;0	6	JP	Cobs_13711	Whole gene duplication	hypothetical_protein_SINV_02031	NO	**YES**
scf0007	5028001	5029001	candidate_367;50;112;-1.161303072;32;351;0;1	32	JP	Cobs_13806	Whole gene duplication		**YES**	NO
scf0007	5029001	5030001	candidate_368;67;154;-1.197783686;34;364;0;1	34	JP	Cobs_13806	Whole gene duplication		**YES**	NO

scf0007	5030001	5031001	candidate_369;49;120;-1.286188148;65;386;356;1	65	JP	Cobs_13802	Whole gene duplication	pol-like_protein	**YES**	NO
scf0007	5092001	5093001	candidate_371;43;91;-1.069968987;18;318;227;1	18	JP	Cobs_13817	Whole gene duplication	hypothetical_protein_EAG_06404	**YES**	NO
scf0007	5093001	5094001	candidate_372;39;97;-1.312569542;16;479;335;1	16	JP	Cobs_13813	Multiple exon duplication	hypothetical_protein_EAG_00248	**YES**	NO
scf0007	5093001	5094001	candidate_372;39;97;-1.312569542;16;479;335;1	16	JP	Cobs_13817	Whole gene duplication	hypothetical_protein_EAG_06404	**YES**	NO
scf0007	5094001	5095001	candidate_373;47;110;-1.212024683;19;997;72;1	19	JP	Cobs_13813	Multiple exon duplication	hypothetical_protein_EAG_00248	**YES**	NO

DELETIONS

Scf	Start	Stop	Name;covBR;covJP;log2ratio;JPhet;exon bases;TE bases;island_binary	Gap (kb)	In	Affected gene	Type	Gene alias	Isl	RNA seq
scf0093	9001	10001	candidate_003;111;58;0.935;0;1000;0;0	0.3	JP	Cobs_14758	Partial exon deletion	g-protein_coupled_receptor_mth2	NO	**YES**
scf0009	261001	262001	candidate_032;59;1;6.659;0;367;0;1	1.8	JP	Cobs_16872	Gene deletion		**YES**	NO
scf0009	466001	467001	candidate_034;77;1;5.689;1;382;195;1	4.6	JP	Cobs_16892	Gene deletion		**YES**	NO
scf0010	88001	89001	candidate_036;124;8;4.020;5;218;104;0	2.4	JP	Cobs_00602	Single exon deletion		NO	NO
scf0010	5244001	5245001	candidate_038;65;0;Inf;0;440;53;0	1.6	JP	Cobs_01070	Gene deletion	major_royal_jelly_protein_1	NO	**YES**
scf0010	5245001	5246001	candidate_039;47;0;9.410;0;426;256;0	1.3	JP	Cobs_01070	Gene deletion	major_royal_jelly_protein_1	NO	**YES**
scf0008	3060001	3061001	candidate_042;44;6;2.880;0;257;247;1	2.7	JP	Cobs_16510	Multiple exon deletion	fatty_acid_synthase	**YES**	NO
scf0003	307001	308001	candidate_044;37;0;10.053;0;324;124;1	1.3	JP	Cobs_14262	Multiple exon deletion		**YES**	NO
scf0003	3279001	3280001	candidate_052;67;22;1.593;2;215;46;1	3.1	JP	Cobs_14454	Gene deletion	hypothetical_protein_SINV_05682	**YES**	NO
scf0003	3288001	3289001	candidate_053;52;6;3.106;0;732;708;1	3.2	JP	Cobs_14460	Gene deletion	integrase_core_domain_protein	**YES**	**YES**
scf0003	3293001	3294001	candidate_054;17;0;5.489;0;346;218;1	4.9	JP	Cobs_14465	Gene deletion	bel12_ag_transposon_polyprotein	**YES**	NO
scf0002	1622001	1623001	candidate_061;59;4;4.011;0;74;70;1	5.9	JP	Cobs_17755	Gene deletion	vitellogenin_receptor	**YES**	NO
scf0002	1623001	1624001	candidate_062;71;6;3.507;1;274;0;1	3.7	JP	Cobs_17755	Gene deletion	vitellogenin_receptor	**YES**	NO
scf0002	1705001	1706001	candidate_063;44;0;7.241;0;287;124;1	4.3	JP	Cobs_17789	Gene deletion	period_circadian_protein	**YES**	**YES**
scf0002	2572001	2573001	candidate_073;48;14;1.840;0;771;225;1	2.4	JP	Cobs_17838	Gene deletion	zinc_finger_mym-type_protein_1-like	**YES**	NO
scf0001	1377001	1378001	candidate_078;77;0;7.584;0;951;0;1	2.0	JP	Cobs_06974	Gene deletion	zinc_knuckle_domain_protein	**YES**	NO
scf0001	1409001	1410001	candidate_083;52;6;3.001;0;180;51;1	0.7	JP	Cobs_06972	Single exon deletion		**YES**	NO
scf0001	1445001	1446001	candidate_085;91;4;4.640;5;120;43;1	3.5	JP	Cobs_06975	Multiple exon deletion	er_degradation-enhancing_alpha-mannosidase-	**YES**	**YES**

								like_3-like		
scf0001	1861001	1862001	candidate_092;56;20;1.470;6;411;254;1	1.8	JP	Cobs_07000	Gene deletion		**YES**	NO
scf0001	4968001	4969001	candidate_106;70;0;12.194;0;521;103;1	3.6	JP	Cobs_07210	Single exon deletion	hypothetical_protein_SINV_09002	**YES**	NO
scf0007	1299001	1300001	candidate_112;31;4;3.044;0;467;100;1	2.5	JP	Cobs_13411	Partial exon deletion	hypothetical_protein_EAG_01487	**YES**	**YES**
scf0007	1423001	1424001	candidate_114;203;5;5.451;3;474;86;1	1.5	JP	Cobs_13414	Gene deletion	hypothetical_protein_SINV_00831	**YES**	NO
scf0007	2576001	2577001	candidate_125;95;0;12.213;0;780;123;1	2.6	JP	Cobs_13524	Gene deletion	tyrosine_partial	**YES**	NO
scf0007	2576001	2577001	candidate_125;95;0;12.213;0;780;123;1	2.3	JP	Cobs_13525	Single exon deletion	hypothetical_protein_EAI_02370	**YES**	NO
scf0007	2905001	2906001	candidate_129;66;16;2.039;0;273;45;1	2.3	JP	Cobs_13563	Single exon deletion	hypothetical_protein_EAG_07634	**YES**	NO
scf0007	5225001	5226001	candidate_130;42;0;11.347;0;616;0;1	0.6	JP	Cobs_13822	Multiple exon deletion	hypothetical_protein_GIP_L7_0050	**YES**	NO
scf0007	5226001	5227001	candidate_131;86;38;1.197;3;242;173;1	3.7	JP	Cobs_13822	Multiple exon deletion	hypothetical_protein_GIP_L7_0050	**YES**	NO
scf0003	2774001	2775001	candidate_288;243;484;-0.995002301;9;325;136;1	5.1	JP	Cobs_14410	Multiple exon deletion	reverse_transcriptase_and_recombinase	**YES**	**YES**

Supplementary Table 8: List of *de novo* assembled contigs containing an ORF

Contig ID	Length	Best blastx hit	Length of hit	Description	E value	Bit score	Frame	Query start	Query end	Hit start	Hit end	Positives	Identical
NODE_388976	14592	XP_003689693	1765	PREDICTED: LOW QUALITY PROTEIN: vitellogenin-like [Apis florea]	0	410	2	3416	5455	251	897	54.60%	35.10%
NODE_372516	1358	EGI70062	895	Transmembrane protein C9orf5 [Acromyrmex echinatior]	1.00E-83	283	1	527	1165	1	212	76.40%	65.70%
NODE_478441	1239	EGI65030	898	Sorting nexin-25 [Acromyrmex echinatior]	1.00E-76	204	2	314	634	31	137	96.30%	92.50%
NODE_426727	2425	EFZ16328	620	hypothetical protein SINV_06913 [Solenopsis invicta]	9.00E-70	190	2	540	1451	352	602	52.80%	44.00%
NODE_417388	855	EFZ16328	620	hypothetical protein SINV_06913 [Solenopsis invicta]	3.00E-56	152	0	424	747	123	231	85.30%	74.30%
NODE_396624	7803	EFN68490	1573	Peripheral-type benzodiazepine receptor-associated protein 1 [Camponotus floridanus]	3.00E-30	141	2	209	538	48	157	74.50%	70.00%
NODE_451872	579	EGI66069	1045	Protein toll [Acromyrmex echinatior]	9.00E-28	117	0	286	516	966	1045	78.80%	75.00%
NODE_241469	369	AEV76939	320	NADH dehydrogenase subunit 1 [Camponotus vafer]	9.00E-24	100	0	1	369	37	159	57.70%	43.90%
NODE_401423	643	XP_003706903	1705	PREDICTED: uncharacterized protein LOC100874905 [Megachile rotundata]	4.00E-20	96.7	2	381	536	34	85	98.10%	88.50%
NODE_443138	611	EGI57979	162	hypothetical protein G5I_13957 [Acromyrmex echinatior]	2.00E-15	78.2	0	135	389	55	142	67.80%	60.00%
NODE_352113	136	EFN84361	94	hypothetical protein EAI_03729 [Harpegnathos saltator]	5.00E-15	69.7	1	1	132	38	81	84.10%	68.20%
NODE_440382	6431	EFZ16363	78	hypothetical protein SINV_09927 [Solenopsis invicta]	8.00E-15	79.7	2	5119	5346	1	78	59.00%	57.70%
NODE_467846	151	EFZ11980	128	hypothetical protein SINV_01763 [Solenopsis invicta]	4.00E-12	63.5	1	1	147	23	71	73.50%	55.10%
NODE_593100	151	EFZ11980	128	hypothetical protein SINV_01763 [Solenopsis invicta]	5.00E-12	63.2	1	5	151	23	71	73.50%	55.10%
NODE_459514	226	EGI65596	271	hypothetical protein G5I_05988 [Acromyrmex echinatior]	8.00E-11	63.5	0	1	126	230	271	83.30%	66.70%
NODE_96922	191	EFN83463	257	hypothetical protein EAI_08541 [Harpegnathos saltator]	2.00E-10	62	0	19	189	136	192	66.70%	52.60%
NODE_144210	300	EFZ09717	207	hypothetical protein SINV_15650 [Solenopsis invicta]	7.00E-10	61.2	1	6	263	9	97	57.80%	38.90%

Supplementary Table 9: List of GO terms underrepresented in TE islands

GO-ID	Term	Category	FDR	#Test	#Ref
GO:0005515	protein binding	F	1.74E-19	48	2533
GO:0016021	integral to membrane	C	4.65E-07	23	1159
GO:0003700	sequence-specific DNA binding transcription factor activity	F	9.48E-07	0	357
GO:0005667	transcription factor complex	C	2.38E-05	1	348
GO:0043565	sequence-specific DNA binding	F	4.50E-05	0	284
GO:0044430	cytoskeletal part	C	1.03E-04	1	317
GO:0007010	cytoskeleton organization	P	1.86E-04	0	250
GO:0006928	cellular component movement	P	2.89E-04	1	291
GO:0005524	ATP binding	F	3.47E-04	17	807
GO:1901566	organonitrogen compound biosynthetic process	P	0.001158612	1	260
GO:0071822	protein complex subunit organization	P	0.001619753	0	207
GO:0065008	regulation of biological quality	P	0.001856809	5	397
GO:0030234	enzyme regulator activity	F	0.002235494	1	245
GO:0050790	regulation of catalytic activity	P	0.002261041	1	248
GO:0015630	microtubule cytoskeleton	C	0.002286604	1	249
GO:0009966	regulation of signal transduction	P	0.004465379	3	307
GO:0009888	tissue development	P	0.004526085	3	310
GO:1901137	carbohydrate derivative biosynthetic process	P	0.004539984	0	182
GO:0007264	small GTPase mediated signal transduction	P	0.004660173	0	185
GO:0015031	protein transport	P	0.004823331	2	274
GO:0007267	cell-cell signaling	P	0.006042638	3	299
GO:0040007	growth	P	0.00628819	0	173
GO:0048667	cell morphogenesis involved in neuron differentiation	P	0.00628819	0	174
GO:0007017	microtubule-based process	P	0.00628819	1	227
GO:0048812	neuron projection morphogenesis	P	0.006382808	0	176
GO:0009887	organ morphogenesis	P	0.008118047	3	293
GO:0090407	organophosphate biosynthetic process	P	0.008811062	0	164
GO:0006468	protein phosphorylation	P	0.009516719	4	323
GO:0046907	intracellular transport	P	0.011008803	3	285
GO:0051128	regulation of cellular component organization	P	0.011653655	1	208
GO:0007276	gamete generation	P	0.012249983	1	212
GO:0015077	monovalent inorganic cation transmembrane transporter activity	F	0.012737248	0	160
GO:0004672	protein kinase activity	F	0.012778601	4	315
GO:0065003	macromolecular complex assembly	P	0.013013762	0	162
GO:0044723	single-organism carbohydrate metabolic process	P	0.013013762	0	162
GO:0048523	negative regulation of cellular process	P	0.015124635	6	372
GO:0005694	chromosome	C	0.01594876	1	200
GO:0009790	embryo development	P	0.01594876	2	236
GO:0034613	cellular protein localization	P	0.01594876	2	238
GO:0022402	cell cycle process	P	0.01594876	2	238
GO:0031090	organelle membrane	C	0.015981543	2	239
GO:0006184	GTP catabolic process	P	0.016245078	2	241

GO:0006091	generation of precursor metabolites and energy	P	0.017699389	0	152
GO:0061061	muscle structure development	P	0.017852228	0	153
GO:0012505	endomembrane system	C	0.018462732	0	155
GO:0044459	plasma membrane part	C	0.02196491	1	190
GO:0007444	imaginal disc development	P	0.022309481	2	233
GO:0008289	lipid binding	F	0.022309481	1	194
GO:0042623	ATPase activity, coupled	F	0.022565122	1	195
GO:0048522	positive regulation of cellular process	P	0.023079374	4	302
GO:0015672	monovalent inorganic cation transport	P	0.02523218	0	145
GO:0009791	post-embryonic development	P	0.030802838	2	226
GO:0006357	regulation of transcription from RNA polymerase II promoter	P	0.031210105	1	187
GO:0048610	cellular process involved in reproduction	P	0.031210105	1	187
GO:0042330	taxis	P	0.035500923	0	134
GO:0009069	serine family amino acid metabolic process	P	0.035923367	3	252
GO:0019226	transmission of nerve impulse	P	0.041678654	2	216
GO:2000026	regulation of multicellular organismal development	P	0.042831962	1	174
GO:0048646	anatomical structure formation involved in morphogenesis	P	0.043495522	1	178
GO:0031981	nuclear lumen	C	0.044951027	6	340

Supplementary Table 10: List of GO terms overrepresented in TE islands

GO-ID	Term	Category	FDR	#Test	#Ref
GO:0003964	RNA-directed DNA polymerase activity	F	7.63E-52	53	16
GO:0006278	RNA-dependent DNA replication	P	7.63E-52	53	16
GO:0015074	DNA integration	P	6.74E-50	48	10
GO:0004190	aspartic-type endopeptidase activity	F	6.46E-15	18	9
**GO:0004984**	**olfactory receptor activity**	**F**	**3.23E-14**	**37**	**106**
**GO:0005549**	**odorant binding**	**F**	**5.00E-13**	**37**	**119**
GO:0003723	RNA binding	F	9.01E-12	64	386
**GO:0050911**	**detection of chemical stimulus involved in sensory perception of smell**	**P**	**6.36E-11**	**24**	**53**
**GO:0007187**	**G-protein coupled receptor signaling pathway, coupled to cyclic nucleotide second messenger**	**P**	**2.62E-06**	**24**	**105**
GO:0006313	transposition, DNA-mediated	P	1.85E-05	7	4
GO:0004803	transposase activity	F	1.85E-05	7	4
GO:0004523	ribonuclease H activity	F	5.06E-05	8	9
GO:0019012	virion	C	7.83E-05	7	6
GO:0003968	RNA-directed RNA polymerase activity	F	3.78E-04	5	2
GO:0004482	mRNA (guanine-N7-)-methyltransferase activity	F	8.49E-04	5	3
**GO:0005835**	**fatty acid synthase complex**	**C**	**0.007123262**	**5**	**7**
GO:0006370	7-methylguanosine mRNA capping	P	0.010608553	5	8
**GO:0016297**	**acyl-[acyl-carrier-protein] hydrolase activity**	**F**	**0.030929857**	**4**	**6**

Supplementary Table 11: GLM of high aggression against intruding workers (intercept = BR x BR)

	Estimate	Std. Error	t value	Pr(>|t|)	
(Intercept)	0.24324	0.04470	5.442	8.91e-08	***
BR x JP	0.05526	0.06485	0.852	0.394568	
BR x *Waur*	0.37995	0.06435	5.904	7.22e-09	***
JP x BR	-0.24324	0.06301	-3.861	0.000131	***
JP x JP	-0.22991	0.06301	-3.649	0.000296	***
JP x *Waur*	0.28378	0.06322	4.489	9.21e-06	***

Null deviance: 87.265 on 433 degrees of freedom
Residual deviance: 63.287 on 428 degrees of freedom
AIC: 410.03

Supplementary Table 12: GLM of high aggression against intruding queens (intercept = BR x BR)

	Estimate	Std. Error	t value	Pr(>|t|)	
(Intercept)	0.546875	0.054254	10.080	< 2e-16	***
BR x JP	-0.005891	0.077664	-0.076	0.94	
JP x BR	-0.412547	0.075863	-5.438	1.23e-07	***
JP x JP	-0.373542	0.073860	-5.057	7.98e-07	***

Null deviance: 59.663 on 266 degrees of freedom
Residual deviance: 49.545 on 263 degrees of freedom
AIC: 317.99

Supplementary Table 13: Quantitative assembly statistics for the raw draft genome assembly

Scaffolded sequence (bp)	182 048 038
N50 scaffold size (bp)	2 570 857
Total number of scaffolds	11 084
N50 contig size (bp)	14 935

Supplementary Table 14: Draft genome sequences from other organisms used for gene annotation or comparative studies

Species	URL
Atta cephalotes	http://antgenomes.org/downloads/acep_scaffolds.fasta.zip<br>http://antgenomes.org/downloads/acep_genome.OGS.1.2.gff.zip
Acromyrmex echinatior	http://antgenomes.org/downloads/aech/Aech_v2.0.fa.gz<br>http://antgenomes.org/downloads/aech/Aech_v3.8.gff.gz
Camponotus floridans	http://antgenomes.org/downloads/cflo_v3.3.fa.zip<br>http://antgenomes.org/downloads/cflo_v3.3.gff.zip
Harpegnathus saltator	http://antgenomes.org/downloads/hsal_v3.3.fa.zip<br>http://antgenomes.org/downloads/hsal_v3.3.gff.zip
Linepithema humile	http://antgenomes.org/downloads/arg_ant_scf4.fasta.zip<br>http://antgenomes.org/downloads/lhum_genome.OGS.1.2.gff.zip
Solenopsis invicta	http://antgenomes.org/downloads/Si_gnF.454scaffolds.fasta.zip<br>http://antgenomes.org/downloads/SI2.2.3.corrected.gff.zip
Pogonomyrmex barbatus	http://antgenomes.org/downloads/pbar_scaffolds_v03.fasta.zip<br>http://antgenomes.org/downloads/pbar_genome.OGS.1.2.gff.zip
Apis mellifera	http://antgenomes.org/downloads/Amel_4.5.AGP.linearScaffold.fa.zip
Nasonia vitripennis	http://antgenomes.org/downloads/Nvit_2.0.linear.fa.zip

Supplementary Table 15: Differences in gene evolution across hymenopteran genomes, based on copy number differences within orthologous groups

	Present in all / missing in one [530]	Single-copy in all / duplicated in one [995]
Cardiocondyla obscurior	78	251
Nasonia vitripennis	98	150
Apis mellifera	56	110
Harpegnathus saltator	50	76
Linepithema humile	18	67
Camponotus floridans	32	67
Pogonomyrmex barbatus	14	65
Solenopsis invicta	155	92
Acromyrmex echinatior	22	30
Atta cephalotes	7	87

Total number of single-copy deletion or duplication event is given in parentheses

Supplementary Table 16: Reads generated per sample in the RNAseq experiment

Sample	Type	Raw read Count	Reads mapped to genes
QUI01	Imago	18 234 979	3 023 851
QUI05	Imago	24 958 424	2 635 246
QUI14	Imago	22 518 877	4 415 260
QUI16	Imago	17 864 237	3 803 684
QUI17	Imago	17 246 589	2 674 107
QUI51	Imago	23 405 068	3 810 613
QUI54	Imago	18 675 375	2 966 886
QUL65	Larva	20 406 972	4 572 339
QUL66	Larva	27 288 687	7 386 863
QUL68	Larva	29 225 761	3 192 075
QUL69	Larva	23 043 865	5 214 123
QUL73	Larva	23 071 947	5 296 047
QUL72	Larva	22 855 327	9 096 679
QUL71	Larva	26 443 413	5 567 634

Supplementary Methods

Organisms

Live colonies of *Cardiocondyla obscurior* were collected from aborted fruits on coconut trees (*Cocos nucifera*) in Brazil (collected in 2009) and from bark cavities in coral trees (*Erythrina* sp.) in Japan (collected in 2010). The colonies were transferred to Regensburg and placed in plastered petri-dishes. Food (honey-soaked shreds of paper; *Drosophila* or small chunks of *Periplaneta americana*) and water were provided every three days and colonies were kept in incubators under constant conditions (12h 28° C light/12h 24° C dark). We emphasize that this is one species because recombinant inbred lines have produced viable offspring for over three years (3-4 generation / year) in our lab. Sampled individuals for subsequent DNA/RNA extractions were transferred to eppendorf tubes, snap-frozen in liquid nitrogen and stored at -80° C.

Colony size

To assess differences in colony structure we used a more recent data set with detailed collection data. Colonies collected and censused immediately in November 2013 (BR) and April 2011 (JP) contained similar numbers of workers (Mann Whitney $U = 778.5$, $Z = -0.634$, $p = 0.526$; BR: median = 28, quartiles 21.75 and 51.25, n = 27 colonies; JP: median = 29, quartiles 16 and 47, n = 64). In contrast queen number was higher in Japan (Mann Whitney $U = 501$, $Z = -3.084$, $p < 0.003$; BR: 5 queens, quartiles 3, 8, n = 27 JP: 10 queens, quartiles 4, 19, n = 64).

Morphometry

We compared body size of workers, queens and males of each population drawn randomly from different source colonies, using four continuous morphological characters (head width (HW), head length (HL), thorax width (TW), and thorax length (TL)), measured under a Keyence VH Z00R. In workers HL and HW were correlated (Pearson's $r = 0.233$, $p = 0.028$, n = 97) as well as TL and TW ($r = 0.257$, $p = 0.012$, n = 96). Workers from the BR lineage had smaller HW (Mann Whitney $U = 394$, $Z = -5.647$, $p < 0.001$) and smaller TW ($U = 36$, $Z = -8179$, $p < 0.001$). In queens all four characters were tightly correlated with each other (minimum Pearson correlation HL – TW, $r = 0.492$, $p < 0.001$, n = 59). Queens from BR and JP did not differ in head size (HW: $U = 378$, $Z = -0.864$, $p = 0.387$; HL: $U = 312$, $Z = -1{,}865$, $p = 0.062$) but BR queens had smaller thoraces (TL: $U = 171$, $Z = -4.003$, $p < 0.001$; TW: $U = 168$, $Z = -4.048$, $p < 0.001$). In wingless males the characters were also strongly correlated (minimum Pearson correlation HL – TW, $r = 0.571$, $p = 0.002$, n = 27) but did not differ between BR and JP (HL: $U = 84$, $Z = -0.340$, $p = 0.756$; TW: $U = 75$, $Z = -0.776$, $p = 0.458$).

Behavioral assays

We tested the behavior of experimental colonies towards individual workers or queens from either the same or the other lineage. Experimental colonies consisted of 20 workers, one mated queen, and brood. Colonies were housed in small petri dishes with plaster flooring and a 5-cent sized deep indentation covered by a dark red cover slide. These colonies were allowed to adjust to their new nest for one week prior to the trials. Trials were performed under dimmed ambient red light (six lux). For each trial we removed the cover slide carefully and waited for five minutes to minimize effects by the disturbance before placing one alien individual into the vicinity of the nest. In addition to workers and mated queens of *C. obscurior*, we also performed trials with individual workers of *Wasmannia auropunctata* (*Waur*), to assess aggression against another ant species. After the introduction, we noted the behavior for a period of 5 minutes or until the intruder was killed. We scored the behavior with 1: Light antennation, 2: Antennation, display of mandible threat, 3: Antennation and short biting/pinches, 4: Antennation, short immobilization and biting, 5: Severe biting, occasional stinging and death of the intruder. Trials for which no interaction between intruder and resident occurred within 5 minutes were discarded. We performed a GLM comparing high aggressive interactions (score 5) versus all other categories combined, separately for workers and queens (Supplementary Table 10-11).

Chemical analysis

We analyzed 8 BR and 8 JP colonies for differences in cuticular lipid profiles. Ants were extracted for 10 min in batches of 6 individuals in 40 µl Hexane containing 30 ng methyl decanoate as internal standard. Extracts were analyzed on a GC2010 gas-chromatograph (GC) connected to a QP2010 plus mass-spectrometer (MS; both Shimadzu, Duisburg, Germany). The GC was equipped with a non-polar capillary column (BPX-5, 30 m length, 0.25 mm inner diameter, 0.25 µm film thickness; SGE Analytical Science, Milton Keynes, UK). Helium was used as carrier gas with a constant linear velocity of 50 cm s^{-1}. The temperature program of the GC-oven started at 80 °C and was raised by 5° C min^{-1} to 300° C. The MS was run in electron impact (EI) mode at 70 eV and set to a scan range from 35 to 600 mz^{-1}. All samples were injected splitless at an injector temperature of 300° C. n-Alkanes were identified by comparing retention times and mass spectra with those of synthetic reference compounds. Methyl-branched CHCs were identified by interpretation of diagnostic ions and comparison of linear retention indices with literature data [1].

For further analysis we used only those peaks that had a minimal area of 1 % in at least 75 % of the samples of at least one lineage. 22 Aitchison-normalized peak areas were subjected to a principal component analysis followed by linear discriminant analysis with leave-one-out cross validation on the first four PCs using the R package vegan [2].

DNA extraction

The reference genome is based on one colony that was kept under strict inbreeding in the lab for four generations prior to extractions. Sampled ants were ground with disposable micro-tube pestles and whole DNA was extracted with CTAB [3]. Extracts were treated with proteinase K and RNAse H, washed twice with ethanol, dried, and finally dissolved in sterile water. We extracted DNA from 900 ants, which were pooled to be sequenced with 454 technology. Extracts of 5, 10 and 30 Brazilian males and 26 Japanese males, respectively were used for Illumina libraries.

DNA library preparation and sequencing

Absorbance measurements at 260 nm and 280 nm (NanoDrop 1000) and Agilent Bioanalyzer traces were obtained for basic quality control of DNA samples designated for paired-end Illumina sequencing. Shearing of extracted DNA was performed on a Covaris S2 AFA system. For Illumina sequencing, we generated 200 and 500 bp insert libraries with Illumina's TruSeq DNA sample preparation kits from 5 µg of total DNA. Quality control and library preparation were carried out by the KFB sequencing centre of the University Regensburg, sequencing runs were performed by Illumina (Hayward, USA) on a HiSeq2000.

Quality control, library preparation, and sequencing of 8 kb and 20 kb long paired end (LPE) libraries (454, Roche) were carried out by Eurofins MWG Operon (Ebersberg, Germany). Extracted DNA was fragmented into the appropriate fragment sizes (8 kb and 20 kb) using the HydroShear DNA Shearing Device (GeneMachine). Further library preparation was performed according to "GS FLX Titanium Paired End Library Prep 20+8kb Span Method Manual" before sequencing on a GS FLX Titanium (Roche).

De novo genome assembly

We generated relatively few genomic 454 reads – about 2.3x genome coverage, a single run of the sequencer. Additional coverage was provided by Illumina reads and connectivity was provided by the 8 kb and 20 kb 454 mate pairs (Supplementary Table 2). The resulting N50 scaffold and contig sizes of the assembly show that the data was sufficient for high quality assembly (Supplementary Table 13). The assembly was created with MSR-CA version 1.4 open source assembler (University of Maryland genome assembly group at ftp://ftp.genome.umd.edu/pub/MSR-CA/). The MSR-CA assembler combines a deBruijn graph strategy with the traditional Overlap-Layout-Consensus employed by various assembly programs for Sanger-based projects (Arachne, PCAP, CABOG, etc.). The MSR-CA uses a modified version of CABOG version 6.1 for contiging and scaffolding. The combined strategy allowed us to natively combine the short 100 bp Illumina reads and longer 454 reads in a single assembly without resorting to an approach that would require one to assemble each type of data

separately and then creating a combined assembly. Total run time for the assembly was approximately 3 days on a 16-core AMD Opteron computer with 128 Gb RAM.

Using CEGMA [4] on the genome sequence to assess the completeness of the assembly, we confirmed complete presence of 244 of 248 ultra-conserved genes (98.39 %). We analysed seven other published ant genomes with CEGMA and they all performed similarly well, with the draft genome of *L. humile* containing the highest number (245) of complete ultra-conserved genes. The other genomes contained 228 (*S. invicta*), 241 (*C. floridanus*), 234 (*A. cephalotes*), 243 (*A. echinatior*), 243 (*P. barbartus*), and 242 (*H. saltator*) complete copies. The percentage of core eukaryotic genes with more than one complete ortholog was elevated in *C. obscurior* (23.77 %), compared to the other analyzed ant genomes (9.65 % - 12.40 %).

Whole RNA extraction, normalized cDNA library preparation, and transcriptome assembly

We sampled individuals from the same BR colony that was used for the genomic DNA sequencing. Whole RNA was extracted from separate pools of eggs, the three larval stages, prepupae, pupal and different adult stages of queens, workers, ergatoid males and winged males using TRIzol (Life technologies) and subsequent Microcon purification (Millipore). Equal quantities of RNA from each extract were combined in a single pool, which was subsequently used to generate a normalized, random-primed cDNA library for emPCR-based sequencing. Sequencing was carried out on a GS FLX using Titanium series chemistry by Eurofins MWG Operon (Ebersberg, Germany), generating 1 245 994 reads (0.4 Gb).

We used the FastX toolkit (http://hannonlab.cshl.edu/fastx_toolkit/) for quality control of raw reads and only kept high quality reads (length 10-550 bases, minimum quality scores of 20 for 70 % of the called bases). The remaining 1 122 247 reads were submitted to the reference based transcriptome assembly with Newbler v2.6 (Roche, options "-cdna -gref -ml 60 -mi 95"). We generated a total of 19 325 contigs ranging between 500 and 12 699 bases length (N50 1 155 bases) that were supplied as EST evidence to MAKER in the subsequent gene annotation.

Gene annotation

MAKER version 2.20 [5] was run on the *C. obscurior* draft genome using the assembled transcriptome, amino acid sequence data from Swiss-prot and the ant genomes portal (Supplementary Table 14), in addition to hand-curated amino acid sequence for desaturase proteins in *Acromyrmex echinatior* and *Pogonomyrmex barbatus* [6]. Repetitive regions were masked using a custom repeat library constructed with RepeatModeler (http://www.repeatmasker.org/RepeatModeler.html), all organisms in Repbase [7], and a list of

known transposable elements in MAKER. *Ab initio* gene predictors (GeneMark [8], Augustus [9], and SNAP [10]) were trained on the assembly and also used by MAKER to generate gene models.

The official gene set contains 17 552 genes, of which 9 552 genes contain a known protein domain as detected by IPRScan [11] and 72.5 % of the genes in the final gene set have an AED (annotation edit distance) of less than 0.5, which is consistent with a well-annotated genome [12]. The total number of *ab silicio* predicted genes falls within the range of recent estimates for the other sequenced ant species [13].

A comparison of the gene set with seven other ant genomes, *Apis mellifera* and *Nasonia vitripennis* using orthologues groups annotated with OrthoDB [14] revealed extensive duplication events in *Cobs* and *Nvit* (Supplementary Table 15).

Functional annotation of Cobs1.4 genes

Gene Ontology (GO) term annotation for Cobs1.4 genes was done using the Blast2GO pipeline [15]. Predicted protein sequences for each gene were blasted against the non-redundant NCBI protein database nr (retrieved May, 3rd 2013) and parsed through Interpro scan (IPS 5-RC6, [11]). Blastx returned hits with e-values less than 1e-10 for ~70 % of the transcripts and 53 818 Interpro domains were annotated in 9 252 gene models. Using Blast2gPipe (v2.5, default settings), 43 166 GO terms were retrieved for 8 908 gene. We also used the Blast2go BDA system to assign provisional gene aliases for 3 415 genes. All computations were performed on the Queen Mary University of London SBCS-informatics Apocrita compute facility.

Repeat annotation

Our goal was to use existing repeat prediction tools to generate *de novo* repeat libraries for several insect genomes. To this end, we implemented a pipeline that has several repetitive element prediction tools at its core.

Our pipeline combines results from RepeatModeler (v1.04) and PILER-DF [16]. RepeatModeler is a wrapper around two *de novo* repetitive element detection algorithms, RECON and RepeatScout. It also uses TandemRepeatsFinder [17] to search for simple repeats and RepeatMasker for masking and annotating repeat elements. PILER-DF is not a part of the RepeatModeler package, but is also used for repeat prediction. For both RepeatModeler and PILER-DF, the output consists of consensus sequences corresponding to repetitive elements in the input genome. Essentially, the repetitive elements found throughout the input genome are clustered into distinct repeat "families" based on similarity of sequence. A repeat "family" sequence can be thought of as a best representative consensus for all of its member sequences. Consensus sequences are then pooled into a repetitive element library for this input genome. In a latter part of the pipeline, we will use our consensus repeat library to scan our genome of interest to find repeat elements.

The first part of the pipeline involves generating repeat family sequences using the tools mentioned above. In the next part of the pipeline we (1) combined RepeatModeler and PILER results, (2) ran quality control, (3) added additional annotations for consensus repeat sequences. Combining the results of multiple prediction tools will inevitably result in duplicates. To remove duplicates, we performed an all-by-all sequence comparison of our combined repeat prediction libraries and retained only one from pairs that show 80% identity over 80% of length (length of shorter sequence).
One pitfall of repeat prediction is that false positives are often genes or gene families containing transposable element-like domains or simple repeat domains (such as the calx-beta motif). When we developed this pipeline using the genomes of various fruit flies, *L. humile* and *A. cephalotes*, we found that the native quality controls in RepeatModeler and PILER did not sufficiently filter false positives. Thus, we enforced a stricter threshold. In our current pipeline, we used the genome of *Drosophila melanogaster* as a reference to find false positives (Blastx hits with at least 50% identity over 50% length), which are removed. We arrived at these Blast parameter thresholds through a combination of Blast searches and manual curation of false positives in several genomes. Although 50/50 is a safe threshold for not including genes into a repeat library, we note that a hard sequence similarity cut-off such as ours serves as a coarse filter.

While the RepeatModeler pipeline annotates its repeat predictions using RepBase [18], PILER has no such functionality. We annotated PILER consensus repeats using RepeatMasker, which uses RepBase as a reference. Additionally, we scanned all consensus repeats for the presence of long terminal repeats (LTR) or terminal inverted repeats (TIR) using custom scripts. After all annotations were updated, the final *C. obscurior* repeat library was output in FASTA and EMBL format.

For predicting repetitive elements in the *C. obscurior* genome, we added a library for *C. obscurior* generated in a first run of the pipeline to our master library consisting of the following: the latest RepBase (at the time, this was version 20121104), our *de novo* consensus repeat libraries generated from the genomes of 7 ants, 6 bees/wasps, and 12 drosophilid flies (see below) and reran the pipeline.

1. Ants – *Atta_cephalotes, Acromyrmex_echinatior, Camponotus_floridanus, Cardiocondyla_obscurior, Harpegnathos_saltator, Linepithema_humile, Pogonomyrmex_barbatus, Solenopsis_invictus*

2. Bees/Wasps – *Apis_florea, Apis_mellifera, Bombus_terrestris, Megachile_rotundata, Nasonia_vitripennis*

3. Flies – *D. ananassae, D. erecta, D. grimshawi, D. melanogaster, D. mojavensis, D. pseudoobscura, D. persimilis, D. sechellia, D. simulans, D. virilis, D. willistoni, D. yakuba*

We used CENSOR [19] for reference-based annotation of repetitive elements in *C. obscurior*. Our master repeat library was used as a reference. CENSOR uses simplistic filters such as seg, xnu, and dust to search for tandem repeats. We supplemented this with our own TandemRepeatsFinder (TRF) results. All hits to our master library were recorded in a GFF3 format file.

Mapping of genomic reads against the Cobs1.4 reference genome

For each lineage, we randomly sampled 140 M 100 bp reads from libraries generated from 26 (JP) and 30 (BR) male pupae. Raw reads were parsed through quality filtration and adapter trimming (Trimmomatic v0.22 (www.usadellab.org/cms/?page=trimmomatic), options: HEADCROP:7 LEADING:28 TRAILING:28 SLIDINGWINDOW:10:10) and mapped against the BR reference genome with BWA samse v0.5.9-r16 [20] in single end mode. Ambiguous reads were re-aligned with Stampy v1.0.21 [21] to reduce misalignments [22]. Aligned reads were stored in sam format.

***De novo* assembly of unmapped reads**

We extracted 23 054 888 Illumina reads generated from the JP lineage that could not be mapped against the reference genome using custom perl scripts. After filtering with Trimmomatic v0.22 (HEADCROP:7 LEADING:28 TRAILING:28 SLIDINGWINDOW:10:10), we generated *de novo* assemblies of these reads using velvetoptimizer v2.2.4 (http://bioinformatics.net.au/software.velvetoptimiser.shtml) with velvet 1.2.07 [23]. The optimised assembly contained 144 664 contigs (N50 8.7 kb, mean length 1.2 kb). We removed short contigs, contigs with extreme coverage, and contigs returning Blastn hits against the BR raw draft assembly with an e-value < 1e-10, leaving a final set of 4 108 contigs (N50 0.4 kb, mean length 0.34 kb) not present in the BR genome assembly. These contigs were blasted (Blastx) against NCBI's non-redundant database (retrieved May, 3rd 2013) and against the Cobs1.4 proteins. Contigs without hits below an e-value of 1e-10 in eukaryotes or with hits against a Cobs1.4 protein (e-value below 1e-10) were removed, producing a set of 17 contigs containing an open-reading frame that are only present in the JP genome.

Calculation of sliding windows

One kb windows of different stats (TEs, exons, SNPs, coverage) were calculated for all scaffolds based on GFF, VCF, and SAM files. For GFF and VCF files, custom bash and perl scripts were used to calculated TE and exon bases per 1 kb, and variant calls (see below) per 1 kb. Coverage per 1 kb was calculated from SAM files, using samtools' depth algorithm [24] and custom bash and perl scripts. Subsequent processing, calculating of 200 kb sliding windows, and plotting of the data was performed with R v3.0.0 (r-project.org).

Detection of small-scale genomic structural variants

To identify differences in the genome affecting genes, we filtered 1 kb windows (see above) where log2 coverage ratio (BR/JP) was below -0.8 or above 0.8. Values below -0.8 suggest either regions of low coverage in BR or regions of elevated coverage in JP, *vice versa* for values above 0.8. We applied a second filter based on exon and TE content of each individual window and selected only those windows containing more annotated exon than TE bases, thus focusing on windows dominated by exonic over transposon sequence. A list of candidate genes was compiled based on intersection of the MAKER annotation with the list of candidate 1 kb windows. The absolute base-wise coverage for BR and JP as well as the log2 coverage ratio were plotted against the genomic position and candidate genes were manually inspected and classified as either partial or full gene deletions or duplications.

Experimental proof-of-principle was conducted by PCR and Sanger sequencing for two deletion candidates (*Cobs_13563* and *Cobs_01070*) and by real-time quantitative PCR for four duplication candidates (*Cobs_13806*, *Cobs_17872*, *Cobs_13486*, and *Cobs_16853*) (see Supplementary Figure 7). For deletion candidates, we designed primers spanning the putative deletion and performed PCR on extracted genomic DNA for both lineages. PCR products were purified and Sanger sequenced to confirm the deletion. For duplication candidates, we designed primers within the putative duplicated genomic sequences and performed qPCR experiments (normalization against a single copy gene (*actin*, *Cobs_04257*)) on genomic DNA, isolated from three different colonies of each population. By calculating the ratio of normalized relative quantities between BR and JP copy number variations were confirmed.

Variant calling

Single nucleotide variant and InDel calling was carried out combining samtools [24] and the GATK [25,26], retaining only those variants called consistently by both tools. Potential PCR duplicates were marked with Picard MarkDuplicates (http://picard.sourceforge.net/). Raw variant calls were produced with the GATK after local realignment around InDels. Subsequently, all calls were annotated and filtered; producing sets of high and low confidence SNVs and InDels,

respectively. The set of high confidence SNVs was used to train the GATK's VariantRecalibrator for variant quality score recalibration to filter additional SNVs from raw variant calls. The final set produced with the GATK consisted of 783 009 called single nucleotide variants and 168 754 InDels.

Raw variant calls produced by samtools were filtered based on mapping quality and genotype quality (Q>29, GQ>31), resulting in a set of 601 214 SNVs and 151 656 InDels.

A total of 567 552 SNVs and 68 430 InDels were called consistently by both tools. The transition from Cobs1.3 to Cobs1.4 removed contaminating endosymbiotic scaffolds, resulting in a final variant set of 553 052 SNVs and 67 987 InDels stored in a single VCF file. Single nucleotide variants were annotated with SNPeff [27] to identify non-synonymous and synonymous substitutions.

Gene Ontology enrichment

To test for enrichment or depletion of certain GO terms in genes in TE islands, we performed a two-tailed GO enrichment analysis. The Gossip package [28], implemented in Blast2go, uses Fisher's Exact Test for each GO term and corrects for multiple testing. GO terms with FDR<0.05 were considered to be significantly enriched/depleted in the test set.

Enrichment of Transposable Element superfamilies

Similarly to the GO enrichment analyses, we tested all TE superfamilies for enrichment in TE islands. We performed one-tailed Fisher's Exact Tests for each superfamily in TE islands, testing for significant enrichment of copy numbers and in a second test for enrichment of total bases compared to other superfamilies in TE islands. We applied FDR corrections for multiple testing and considered all TE superfamilies to be significantly enriched in copy number or base count with an FDR<0.05.

Gene expression analysis with RNAseq

We extracted whole RNA from 7 individual mated queens of the same age (4 weeks after pupal molt) and 7 individual developing queens in the early 3rd larval instar (11-13 days). To sample larvae, we set up experimental colonies consisting of 20 workers and 20 to 30 methoprene-treated eggs, as queen development can be induced by treatment with low concentrations of the JH-analogue [29]. Unsampled larvae from these colonies were kept alive to confirm the exclusive development of queen pupae. Sampled queens and larvae were placed individually in 1.5 ml eppendorf tubes, snap-frozen in liquid nitrogen, and kept at -80° C till further processing.

We extracted whole RNA with the RNeasy Plus Micro kit (Qiagen) yielding 27 to 153 ng per individual larvae and 57 to 122 ng per individual queen. Single end Illumina libraries from amplified RNA (Ovation RNAseq system V2) were generated following the manufacturers protocol (Ovation Rapid Multiplexsystem, NuGEN). Sequencing on an Illumina HiSeq1000 at the in-house sequencing centre (KFB, Regensburg, Germany) generated ~20 M 100 bp reads per sample (Supplementary Table 16). Raw reads were filtered for adapter contamination (cutadapt, [30]), parsed through quality filtration (Trimmomatic v0.27, options: LEADING:10 TRAILING:10 SLIDING:4:10 MINLEN:15), and mapped against the reference genome using the tophat2 (v2.0.8) and bowtie2 (v2.1.0) package ([31,32], --b2-sensitive mode, mapping rate ~50 %). Low mapping rates are most likely a consequence of the required amplification step during library preparation. Gene expression analysis was carried out with DESeq2 [33], based on count tables produced with HTSeq [34] against the Cobs1.4 MAKER annotation (Supplementary Table 16). Genes were considered to be differentially expressed at an FDR < 0.05 and expression values are reported as untransformed base means of read counts per treatment group, after correcting for library size differences ("size factor normalization").

Calculation of exon wide CpG o/e values

Observed to expected CpG values for all exons were calculated as [35]:

$$\frac{Obs}{Exp} CpG = \frac{n_{CpG}}{n_C \times n_G} \times N \qquad (1)$$

where N is the total number of nucleotides in the analysed exon.

Supplementary References

1. Carlson, D. A., Bernier, U. R. & Sutton, B. D. Elution patterns from capillary GC for methyl-branched alkanes. *J. Chem. Ecol.* **24,** 1845–1865 (1998).
2. Oksanen, J. *et al.* Package 'vegan'. (2013).
3. Sambrook, J. & Russell, D. W. *Molecular Cloning*. (CSHL Press, 2001).
4. Parra, G., Bradnam, K. & Korf, I. CEGMA: a pipeline to accurately annotate core genes in eukaryotic genomes. *Bioinformatics* **23,** 1061–1067 (2007).
5. Cantarel, B. L. *et al.* MAKER: An easy-to-use annotation pipeline designed for emerging model organism genomes. *Genome Res.* **18,** 188–196 (2007).
6. Simola, D. F. *et al.* Social insect genomes exhibit dramatic evolution in gene composition and regulation while preserving regulatory features linked to sociality. *Genome Res.* **23,** 1235-1247 (2013).
7. Jurka, J. *et al.* Repbase Update, a database of eukaryotic repetitive elements. *Cytogenet. Genome Res.* **110,** 462–467 (2005).
8. Ter-Hovhannisyan, V., Lomsadze, A., Chernoff, Y. O. & Borodovsky, M. Gene prediction in novel fungal genomes using an ab initio algorithm with unsupervised training. *Genome Res.* **18,** 1979–1990 (2008).
9. Stanke, M., Schöffmann, O., Morgenstern, B. & Waack, S. Gene prediction in eukaryotes with a generalized hidden Markov model that uses hints from external sources. *BMC Bioinformatics* **7,** 62 (2006).
10. Korf, I. Gene finding in novel genomes. *BMC Bioinformatics* **5,** 59 (2004).
11. Quevillon, E. *et al.* InterProScan: protein domains identifier. *Nucleic Acids Res.* **33,** W116–W120 (2005).
12. Holt, C. & Yandell, M. MAKER2: an annotation pipeline and genome-database management tool for second-generation genome projects. *BMC Bioinformatics* **12,** 491–491 (2010).
13. Wissler, L., Gadau, J., Simola, D. F., Helmkampf, M. & Bornberg-Bauer, E. Mechanisms and dynamics of orphan gene emergence in insect genomes. *Genome Biol. Evol.* **5,** 439-455 (2013).
14. Waterhouse, R. M., Tegenfeldt, F., Li, J., Zdobnov, E. M., Kriventseva, E. V. OrthoDB: a hierarchical catalog of animal, fungal and bacterial orthologs. *Nucleic Acids Res.* **41,** D358–65 (2013).
15. Conesa, A. & Götz, S. Blast2GO: A Comprehensive Suite for Functional Analysis in Plant Genomics. *Int. J. Plant Genomics* **2008,** 1–12 (2008).
16. Edgar, R. C. & Myers, E. W. PILER: identification and classification of genomic repeats. *Bioinformatics* **21,** i152–i158 (2005).
17. Benson, G. Tandem repeats finder: a program to analyze DNA sequences. *Nucleic Acids Res.* **27,** 573–580 (1999).
18. Smith, C. D. *et al.* Improved repeat identification and masking in Dipterans. *Gene* **389,** 1–9 (2007).
19. Kohany, O., Gentles, A. J., Hankus, L. & Jurka, J. Annotation, submission and screening of repetitive elements in Repbase: RepbaseSubmitter and Censor. *BMC Bioinformatics* **7,** 474 (2006).
20. Li, H. & Durbin, R. Fast and accurate long-read alignment with Burrows-Wheeler transform. *Bioinformatics* **26,** 589–595 (2010).
21. Lunter, G. & Goodson, M. Stampy: A statistical algorithm for sensitive and fast mapping of Illumina sequence reads. *Genome Res.* **21,** 936–939 (2011).
22. Nielsen, R., Paul, J. S., Albrechtsen, A. & Song, Y. S. Genotype and SNP calling from next-generation sequencing data. *Nat. Rev. Gen.* **12,** 443–451 (2011).
23. Zerbino, D. R. & Birney, E. Velvet: Algorithms for de novo short read assembly using de Bruijn graphs. *Genome Res.* **18,** 821–829 (2008).
24. Li, H. A statistical framework for SNP calling, mutation discovery, association mapping and population genetical parameter estimation from sequencing data. *Bioinformatics* **27,** 2987–2993 (2011).
25. McKenna, A. *et al.* The Genome Analysis Toolkit: A MapReduce framework for analyzing next-generation DNA sequencing data. *Genome Res.* **20,** 1297–1303 (2010).
26. DePristo, M. A. *et al.* A framework for variation discovery and genotyping using next-generation DNA sequencing data. *Nat. Genet.* **43,** 491–498 (2011).
27. Cingolani, P. *et al.* A program for annotating and predicting the effects of single nucleotide polymorphisms, SnpEff: SNPs in the genome of *Drosophila melanogaster* strain w1118; iso-2; iso-3. *Fly* **6,** 80–92 (2012).
28. Blüthgen, N. *et al.* Biological profiling of gene groups utilizing Gene Ontology. *Genome Inform.* **16,** 106–115 (2005).
29. Schrempf, A. & Heinze, J. Proximate mechanisms of male morph determination in the ant

Cardiocondyla obscurior. *Evol. Dev.* **8,** 266–272 (2006).
30. Martin, M. Cutadapt removes adapter sequences from high-throughput sequencing reads. *EMBnet journal* (2011).
31. Kim, D. *et al.* TopHat2: accurate alignment of transcriptomes in the presence of insertions, deletions and gene fusions. *Genome Biol.* **14,** R36 (2013).
32. Langmead, B. & Salzberg, S. L. Fast gapped-read alignment with Bowtie 2. *Nat. Methods* **9,** 357–359 (2012).
33. Love, M. I., Huber, W. & Anders, S. Moderated estimation of fold change and dispersion for RNA-Seq data with DESeq2. *bioRxiv* (2014).
34. Anders, S. HTSeq: Analysing high-throughput sequencing data with Python. *URL http://www-huber. embl. de/users/anders/HTSeq/doc/overview. html* (2010).
35. Glastad, K. M., Hunt, B. G. & Goodisman, M. A. D. Evidence of a conserved functional role for DNA methylation in termites. *Insect Mol. Biol.* **22,** 143–154 (2012).
